# ARID3B Directly Regulates Ovarian Cancer Promoting Genes

**DOI:** 10.1371/journal.pone.0131961

**Published:** 2015-06-29

**Authors:** Alexander Bobbs, Katrina Gellerman, William Morgan Hallas, Stancy Joseph, Chao Yang, Jeffrey Kurkewich, Karen D. Cowden Dahl

**Affiliations:** 1 Department of Biochemistry and Molecular Biology, Indiana University School of Medicine-South Bend, South Bend, Indiana, United States of America; 2 Harper Cancer Research Institute, South Bend, Indiana, United States of America; 3 Department of Chemistry and Biochemistry, University of Notre Dame, Notre Dame, Indiana, United States of America; 4 Department of Biological Sciences, University of Notre Dame, Notre Dame, Indiana, United States of America; 5 Indiana University Melvin and Bren Simon Cancer Center, Indianapolis, Indiana, United States of America; Saint Louis University School of Medicine, UNITED STATES

## Abstract

The DNA-binding protein AT-Rich Interactive Domain 3B (ARID3B) is elevated in ovarian cancer and increases tumor growth in a xenograft model of ovarian cancer. However, relatively little is known about ARID3B's function. In this study we perform the first genome wide screen for ARID3B direct target genes and ARID3B regulated pathways. We identified and confirmed numerous ARID3B target genes by chromatin immunoprecipitation (ChIP) followed by microarray and quantitative RT-PCR. Using motif-finding algorithms, we characterized a binding site for ARID3B, which is similar to the previously known site for the ARID3B paralogue ARID3A. Functionality of this predicted site was demonstrated by ChIP analysis. We next demonstrated that ARID3B induces expression of its targets in ovarian cancer cell lines. We validated that ARID3B binds to an epidermal growth factor receptor (EGFR) enhancer and increases mRNA expression. ARID3B also binds to the promoter of Wnt5A and its receptor FZD5. FZD5 is highly expressed in ovarian cancer cell lines, and is upregulated by exogenous ARID3B. Both ARID3B and FZD5 expression increase adhesion to extracellular matrix (ECM) components including collagen IV, fibronectin and vitronectin. ARID3B-increased adhesion to collagens II and IV require FZD5. This study directly demonstrates that ARID3B binds target genes in a sequence-specific manner, resulting in increased gene expression. Furthermore, our data indicate that ARID3B regulation of direct target genes in the Wnt pathway promotes adhesion of ovarian cancer cells.

## Introduction

In the United States, ovarian cancer is the 5^th^ most common cancer in women and the most lethal gynecological cancer. In 2014, it is expected that there have been 21,980 new cases of ovarian cancer, and 14,270 deaths [1]. We demonstrated that the DNA-binding protein ARID3B is overexpressed in serous ovarian cancer; ARID3B’s expression in the nucleus correlates with disease relapse [2, 3]. The goal of this study was to mechanistically identify direct target genes of ARID3B that may contribute to ovarian cancer progression.

ARID3B belongs to a family of AT-Rich Interactive Domain (ARID) proteins that are involved in chromatin remodeling and regulation of gene expression. These proteins are characterized by the ARID DNA-binding domain, a highly conserved sequence of ~100 amino acids [4]. ARID3B has an ARID domain that shares 89.9% amino acid identity with its paralogue ARID3A (a B-cell activator originally named "Bright") that has a binding consensus site of "AATTAA" [5–7]. Mobility shift assays have shown that ARID3B can bind Matrix Attachment Regions that are also bound by ARID3A from IgH [8]. Recently it was reported that ARID3B binds to the Oct4 promoter and regulates its expression, however, an unbiased approach to identify direct ARID3B target genes has not been reported [9].

ARID proteins are involved in development and tissue-specific gene expression, and aberrant expression has been associated with tumorigenesis [10]. *Arid3b* is an essential gene; null embryos die mid-gestation, exhibiting severe defects in development of the heart, neural tissue, craniofacial structures, limb buds, and formation of the apical endodermal ridge [11–13]. ARID3B is overexpressed in neuroblastoma, particularly stage IV tumors, and cooperates with MYCN to increase oncogenic potential and proliferation [14, 15]. In serous ovarian cancer, ARID3B is elevated [2]. Nuclear expression of ARID3B correlates with disease recurrence [3]. Furthermore, overexpression of ARID3B in ovarian cancer cells accelerates tumor growth in a xenograft model of ovarian cancer [3]. The target genes that are regulated by ARID3B and the molecular mechanisms by which ARID3B impacts tumorigenesis in ovarian cancer are not known.

In this study we identified direct gene targets of ARID3B in ovarian cancer cells through Chromatin Immunoprecipitation (ChIP) followed by microarray (ChIP-Chip) technology. The binding regions of ARID3B were characterized by computational bioinformatic analysis and yielded a highly conserved binding site. Among the target genes of ARID3B are members of the EGFR, NOTCH, TNF, and Wnt signaling pathways. We were particularly interested in ARID3B's effect on the Wnt signaling pathway because ARID3B has binding regions in four Wnt pathway genes: WNT5A, FZD5, APC, and MYC. WNT5A and FZD5 are overexpressed in ovarian cancer and correlate with poor prognosis, and Wnt activity is known to regulate cell proliferation and death [16–19]. Upregulation of FZD5 and the ligand WNT7A increase tumor growth and cell adhesion [20]. We found that ARID3B increases expression of FZD5, APC, and MYC. Overexpression of FZD5 or ARID3B in ovarian cancer cells increases adhesion to several ECM proteins, including fibronectin and vitronectin, while knockdown of FZD5 or editing of ARID3B causes a loss of adhesion to certain ECM components. Additionally, knockdown of FZD5 in cells where ARID3B is overexpressed leads to decreased adhesion and decreased ARID3B induced adhesion to collagen II, collagen IV, and tenascin. These results suggest that direct regulation of Wnt signaling by ARID3B may contribute to ovarian cancer progression.

## Materials and Methods

### Cell Culture

**Fig 1:**
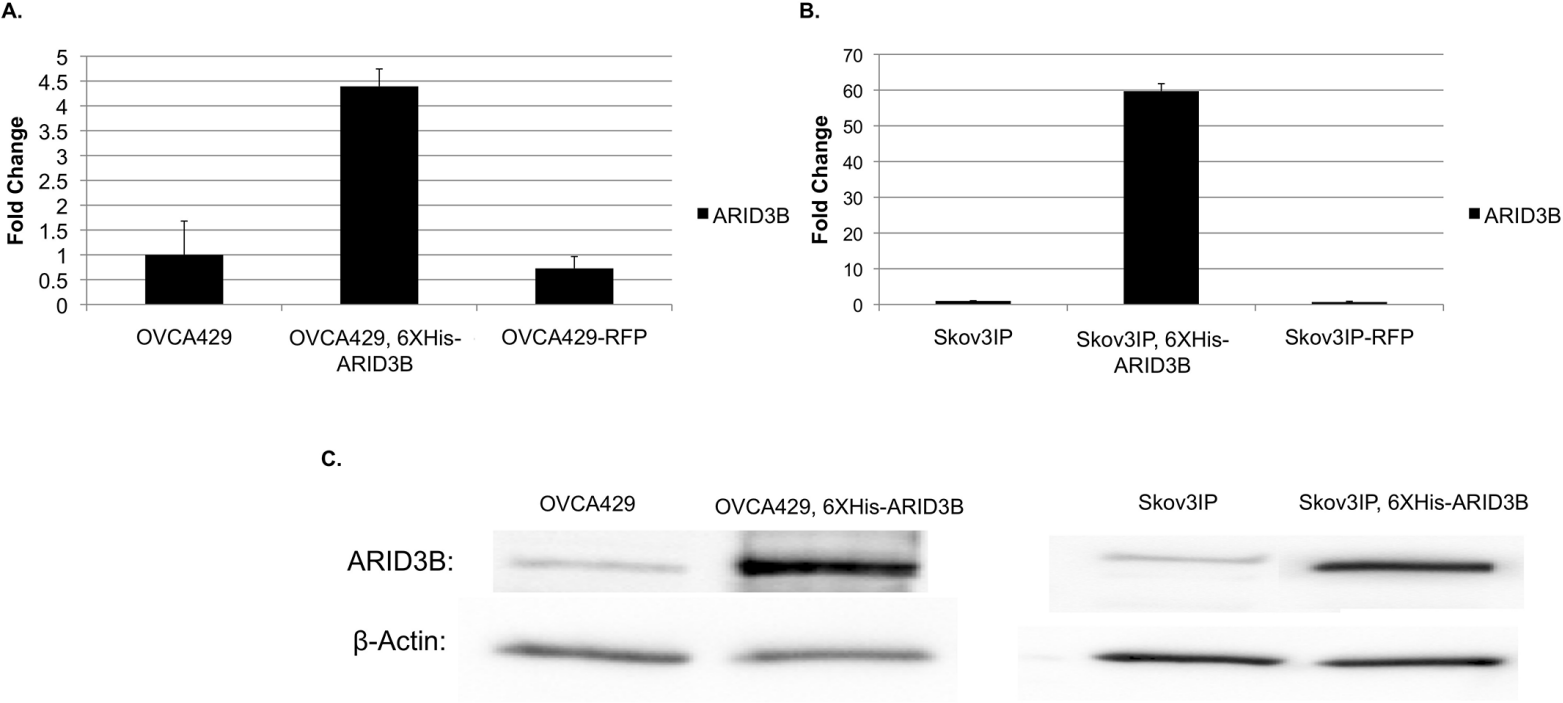
ARID3B expression in ovarian cancer cell lines. ARID3B expression was assessed in parental, RFP-expressing, and 6XHis-ARID3B OVCA429 (A) and Skov3IP (B) cell lines, using both qRT-PCR. (C) A western blot of parental and ARID3B-expressing cell lines confirms this result.

Cell lines were grown at 37°C with 5% CO_2_. OVCA429 cells (provided by Dr. Bast, MD Anderson Cancer Center, Houston, TX and described in [21]) were grown in Minimal Essential Medium (MEM). We obtained Skov3IP cells from Dr. Mills, MD Anderson Cancer Center, Houston, TX. The derivation of Skov3IP cells is described in Yu et al [22]. Skov3IP cells were grown in McCoys Media 5A. Media was supplemented with 10% fetal bovine serum (FBS) (Atlas, Ft. Collins, CO), 0.1 mM L-glutamine, 1mM sodium pyruvate, 50 U/mL penicillin, and 50 μg/mL streptomycin. OVCA429 and Skov3IP cells expressing ARID3B, FZD5 (pLenti-C-mGFP, Origene, Rockville, MD), or control Red Fluorescent Protein (RFP) were created as previously described and levels of ARID3B overexpression were similar to those achieved in our previous studies (Fig 1) [23]. Editing of the ARID3B gene in OVCA429 cells was accomplished using transfected sgRNA/Cas9 all-in-one expression vector targeting ARID3B (p-CRISPR-CG01, Geneocopeia, Rockville, MD). As a control for CRISPR-edited cell lines, OVCA429 cells were transduced with a Cas9 nuclease expression clone (CP-LvC9NU-01, Geneocopeia, Rockville, MD) and a scrambled sgRNA control vector (p-CRISPR-LvSG02, Geneocopeia, Rockville, MD). For FZD5 transduction, Skov3 cells (ATCC, Manassas, VA, USA, #HTB-77) [24] were used in place of Skov3IP cells since our SKOV3IP cells express GFP and the FZD5 expression vector expresses GFP (pLenti-C-mGFP). FZD5 knock-down was achieved using a transduced shRNA vector (pGFP-C-shLenti, Origene, Rockville, MD). OVCAR3 cells (ATCC, #HTB-161) [25] were grown in RPMI media with 20% FBS and 10mg/mL insulin. CAOV3 (ATCC, #HTB-75) [26] and 293FT (Life Technologies, Carlsbad, CA, #R700-07) [27] cells were grown in Dubecco's Modified Eagle Medium (DMEM) with 10% FBS. IOSE398 cells (from Dr. Stack, Harper Cancer Research Institute, Notre Dame, IN described in [28]) were grown in a 1:1 mixture of Media 199 and MCDB105, with 5% FBS and 50 μg/mL gentamicin. All cell culture reagents except for the FBS were from Invitrogen (Carlsbad, CA). Cell lines were authenticated on October 1, 2013, at ATCC by STR profiling.

### Xenograft mouse models of ovarian cancer

All studies were approved by the University of Notre Dame IACUC committee (Protocol 14–060) and were conducted in accordance with the guidelines of the US Public Health Service Policy for the Humane Care and Use of Laboratory Animals. Six-week-old female nude nu/nu mice (Charles River, Wilmington, MA) were maintained at the Freimann Life Science Center (University of Notre Dame). In the pilot study (4 mice per group), 1x10^6^ SKOV3IP-RFP or SKOV3IP-ARID3B cells in 200 μl of phosphate buffered saline (PBS; 137 mM NaCl, 2.7 mM KCl, and 11.9 mM phosphate buffer, pH 7.4) were injected intraperitoneally (IP) into nude mice. Mice were monitored weekly for tumor growth (with a ventral and dorsal image each week). The growth of tumors and imaging is reported in Roy, L., et al[3]. Since the IP tumor growth results in wide spread tumor dissemination, it is hard to accurately quantitate tumor volume for multiple tumors *in vivo*. Mice were euthanized when by *in vivo* imaging detected multiple large tumors or if mice showed any signs of illness including a distended abdomen.

### Microarray Analysis and Bioinformatics

Three RNA samples each from SKOV3IP-RFP and SKOV3IP-ARID3B ascites fluid/peritoneal washes were prepared [3]. The microarray was performed at the Notre Dame Genomics Core Facility on an Affymetrix Human Genome U133 Plus 2 GeneChip (Affymetrix, Santa Clara, CA). The probe set expression levels were normalized and summarized by using the GC robust multi-array average (GCRMA) algorithm [29]. Control probe sets and probe sets that were “not detectable” were filtered out for further analysis. “Not detectable” was defined as probe sets that are either being called “absent” by Affymetrix's Call Detection Algorithm (Manual titled “GeneChip Expression Analysis Data Analysis Fundamentals”, Affymetrix; www.affymetrix.com) in all of the six arrays, or having all its expression reported by GCRMA less than 2.5. Significance Analysis of Microarrays (SAM,[30]) was used for detecting the differentially expressed genes between the ARID3B and RFP controls. One thousand twelve probe sets were found to be significant with a false discovery rate (FDR) less than 10%. Of these probe sets, 199 were down regulated by ARID3B. A second, more stringent analysis normalized the raw microarray data by RMA, and required a FDR of less than 5%. Pathway analysis was conducted by cross-referencing regulated genes with their associated Gene Ontology (GO) terms listed in the Ensembl database.

### Chromatin immunoprecipitation followed by microarray (ChIP-Chip)

The ChIP-Chip procedure was performed according to the protocol of Tamimi et. Al. [31]. Briefly, Ni^2+^-NTA magnetic beads were used to isolate sheared chromatin complexes containing the (His)-6-ARID3B fusion protein from lysates. A positive control (100% input, no Ni^2+^-NTA) and negative control (H2O plus Ni^2+^-NTA added to sample) were also included. Microarray hybridization was performed using a NimbleGen Human 2.1M Deluxe Promoter Array following manufacturer's instructions as described below. Sample integrity for experimental ChIP and control input samples was verified with an Agilent Bioanalyzer (Agilent Technologies, Inc., Santa Clara, CA). One μg of experimental and control samples were labeled with cy3 and cy5-labeled random nonamers, respectively (Roche NimbleGen, Inc., Madison, WI). A 34 μg aliquot of each cy-dye labeled product was pooled and hybridized to a microarray at 42°C for 16–20 hours. Microarrays were washed, dried, and then scanned in a NimbleGen MS200 scanner at 2 μm resolution. Microarray images were visualized and analyzed with NimbleScan software v2.5 (Roche NimbleGen, Inc.).

Calculation of the ARID3B binding site was accomplished using both MEME (University of Queensland, Brisbane, Australia [32, 33] and ALLEGRO (Tel Aviv University, Tel Aviv, Israel) [34, 35]. Sequence data of ARID3B binding regions as determined by our ChIP-Chip experiments were extracted using a custom-built perlscript. The top 50 ARID3B binding site sequences were scanned for common motifs using MEME. In a parallel analysis, all significant ARID3B binding site sequences were compared against a background genome using ALLEGRO.

### Chromatin Immunoprecipitation (ChIP)

Sheared chromatin was prepared from 90% confluent ovarian cancer cells using the Pierce Agarose ChIP Kit (Rockford, IL) and a published protocol from Cold Spring Harbor [36]. DNA shearing was accomplished using an EpiShear Probe Sonicator (Active Motif, Carlsbad, CA), with a series of 10 20-second pulses at 25% amplitude. Fifty μg of sheared chromatin was used for each immunoprecipitation (IP). IP was performed using an anti-ARID3B antibody (Bethyl Laboratories, A302-564A, Montgomery, TX) or IgG (Pierce, Rockford, IL) and ProteinA-Agarose magnetic beads. A sample of "Input DNA" was collected before IP for normalization. ChIP samples were reverse-crosslinked by heating at 65°C for 4 hours in the presence of 20 μg Proteinase K and 250 mM NaCl, and cleaned up using a standard phenol/chloroform extraction followed by ethanol precipitation.

ChIP DNA samples were analyzed with quantitative polymerase chain reaction (qPCR), using Sso Fast EvaGreen Supermix (Bio-Rad, Hercules, CA). Each ChIP DNA sample was compared to the appropriate Input DNA sample. Primers were purchased from Integrated DNA Technologies (Coralville, IA), and designed to flank proposed ARID3B binding sites:

EGFR F: CTCAAGTGTCTCATACTACC / R: GTCATTGGGCAAACCACTG

BTC F: GTCTCAGCCTCCCAAGTAGC / R: CTAACAGGTATAATGTCACAG

WNT5A F: AGTGATTCTCCTGCCTCAGC / R: TGGAAGGGATGAATTTGGTC

MYC F: GGAGGCCAGATGCATGAG / R: TAC CTA TGG CTG TTA GAA TC

FZD5 F: GCACAATGGCTCATGCTTG / R: CGCAATCTTGGCTCACTGC

APC F: CTCCTGACCTCAAGTGATCC / R: CAGTCACTGCTTATAGAATC

RIPK1 F: GTCTTGAACTCCTGACCTCG /

R: ACAGAAACTCCATGCAAACC

NOTCH2 F: GTCAGGAGTTTGAGACC / R: ACTGCAATCTCTGCCTCC

NUSAP1 F: TTCACATGCCTCATTAAGAG / R: TCCCGAGTAGCTGGGATTCC

CASP1 F: ACTCAAGCAATTCACTCACG / R: CATTCTGAGTCCAGAGCCTG

**LPAR1** **F: TGAAGAGTTGCGTATTAAC / R: AGTTTCATGGGTGCTATACC**

CENPN F: ATCTACTGTATGTCAGACAC / R: CAGGCACCCGATACCACG

CEP55: F: GCAGGAGTTCGTGATCAGAG / R: CCAGCTAATTCTGGGATCG

### Gene Expression

RNA from OVCA429 and Skov3IP cells was isolated using TRIzol (Invitrogen, Carlsbad, CA), according to the manufacturer's instructions. Complementary DNA (cDNA) was prepared from 500 ng of RNA using High Capacity cDNA Reverse Transcription Kit (Life Technologies, Waltham, MA) as directed. Reactions were run either using iTaq Universal Probes Supermix (Bio-Rad) or Sso Fast EvaGreen Supermix (Bio-Rad). All gene expression primer sets were obtained from Integrated DNA Technologies (Coralville, IA), with the exception of ARID3B (from Life Technologies, Carlsbad, CA). quantitative reverse transcribed polymerase chain reaction (qRT-PCR) reactions were run in triplicate and normalized to expression of GAPDH. Primer Assays are as follows: APC: Hs.PT.56a.3539689, ARID3B: HS01084949_g1, BTC: Hs.PT.56a.3511718, CASP1: Hs.PT.56a.39699622, CENPK: Hs.PT.56a.40187080.g, CENPN: Hs.PT.56a.22431568.g, CEP55: Hs.PT.56a.279272.g, EGFR: Hs.PT56a.20590781, FZD5: Hs.PT.56a.3585264, GAPDH: Hs.PT.39a.22214836, MYC: Hs.PT.49a.3659201.g, NOTCH2: Hs.PT.512811432, NUSAP: Hs. PT.51.21077614, WNT5A: Hs.PT.56a.22221435.

### Western Blot

Whole-cell protein lysates were obtained by lysing OVCA429 and Skov3IP ovarian cancer cells in RIPA buffer (50 mM Tris pH 7.5, 150 mM NaCl, 1% NP-40, 0.5% EDTA, 0.1% SDS, and 1X Halt Protease & Phosphatase Inhibitor Cocktail (Pierce, Rockford, IL)). Protein concentration was measured using a Bicinchoninic Acid (BCA) assay according to standard protocol (Pierce, Rockford, IL). Proteins were detected using the following antibodies: ARID3B (#A302-564A, Bethyl Laboratories, Montgomery, TX, Rabbit Polyclonal, Synthetic Peptide Antigen), FZD5 (#MC-4273, MBL, Woburn, MA, Rabbit Polyclonal, Synthetic Peptide Antigen), β-actin (#AM1829b, Abgent, San Diego, CA, Mouse Monoclonal, Recombinant Protein Antigen), GAPDH (#ab128915, Abcam, Cambridge, England, Rabbit Monoclonal, Synthetic Peptide Antigen), COXIV (#4850, Cell Signaling Technology, Rabbit polycolonal, synthetic peptide corresponding to residues surrounding Lys29 of human COXIV), and ARID3A (Rabbit polyclonal antibody, a kind gift from H. Tucker UT Austin) followed by a secondary anti-rabbit HRP-conjugated antibody (GE Health Care, Knox, IN). Imaging and quantitation were conducted using a Bio-Rad Chemidoc XRS+ System, running Imager Lab Software (Hercules, CA).

### ECM Adhesion Assay

Cellular attachment to ECM components was determined using a Colorimetric ECM Cell Adhesion Array Kit (Millipore, Billerica, MA). ECM attachment assays were performed on OVCA429 and Skov3 cells expressing ARID3B, FZD5, FZD5 shRNA, or containing CRISPR-edited ARID3B. After 1 hour of incubation, non-adhering cells were washed away, with the remaining adherent cells stained according to the manufacturer's directions. Stained cells were washed with water, and the stain was solubilized with the kit extraction buffer. Light absorption at 540 nm was measured on a Spectramax Plus (Molecular Devices, Sunnyvale, CA). To calculate differences in cellular adhesion, absorption measurements were reported as a fold change over OVCA429-RFP or Skov3-RFP adhesion for each ECM component.

### TCF/Lef reporter assay

We transfected 293FT cells grown to 80% confluency in a 12-well plate, using Lipfectamine 2000 (Invitrogen, Carlsbad, CA), with 500 ng of DNA. All cells were transfected with TOPflash or FOPflash reporter plasmids alongside a vector force-expressing ARID3B (pLenti-suCMV-Rsv, Gentarget, San Diego, CA), FZD5 (pLenti-C-mGFP, Origene, Rockville, MD) Wnt5a (pLenti-C-mGFP), or an empty pLenti-C-mGFP vector. All samples were also cotransfected with a Renilla luciferase control to normalize for cell number and transfection efficiency. Luciferase activity was measured using a Promega Dual Luciferase Assay Kit (Promega, Madison, WI) according to the manufacturer's instructions and measured on a TD 20/20 luminometer (Turner Designs, Sunnyvale, CA). A similar experiment was conducted with 3T3 "Leading Light" cells (Enzo Life Sciences, Farmingdale, NY). As these cells express a Wnt Reporter luciferase, they were only transfected with the above-mentioned ARID3B, FZD5, and Wnt5a vectors, and normalized by protein concentration. All conditions were run in triplicate and normalized to the Renilla internal control.

### Statistics

qPCR data and cell adhesion t-statistics were calculated using the Smith-Satterthwaite procedure with unequal population variances. Statistical significance was assigned to comparisons with a p-value of 0.05 or lower. Over-representation of Gene Ontology (GO) terms was calculated using a Chi-Squared Test.

## Results

### ARID3B binds regulatory regions of DNA

To gain insight into how ARID3B regulates ovarian tumor growth we sought to identify ARID3B regulated genes. Our first step was to identify regulatory regions (such as promoters and enhancers) bound by ARID3B via ChIP-Chip analysis in ovarian cancer cells overexpressing ARID3B (6XHis-ARID3B). OVCA429 cells were transduced with ARID3B as previously described [23]. Since the expression levels of ARID3B in the cells used for the ChIP-Chip were very high (about a 300-fold increase) we chose to validate genes at more moderate levels of ARID3B expression that are more likely to be encountered *in vivo* (Fig 1). Sheared chromatin complexes bound to His-tagged ARID3B were isolated using Ni^2+^-NTA magnetic beads, then hybridized to a NimbleGen Human 2.1M Deluxe Promoter Array. Statistical analysis of the ChIP-Chip array revealed 2,367 genomic regions bound by ARID3B (S1 Table). The genomic regions identified by ChIP-Chip ranged from 397 base pairs to 3,198 base pairs (median: 1,131 base pairs).

**Fig 2:**
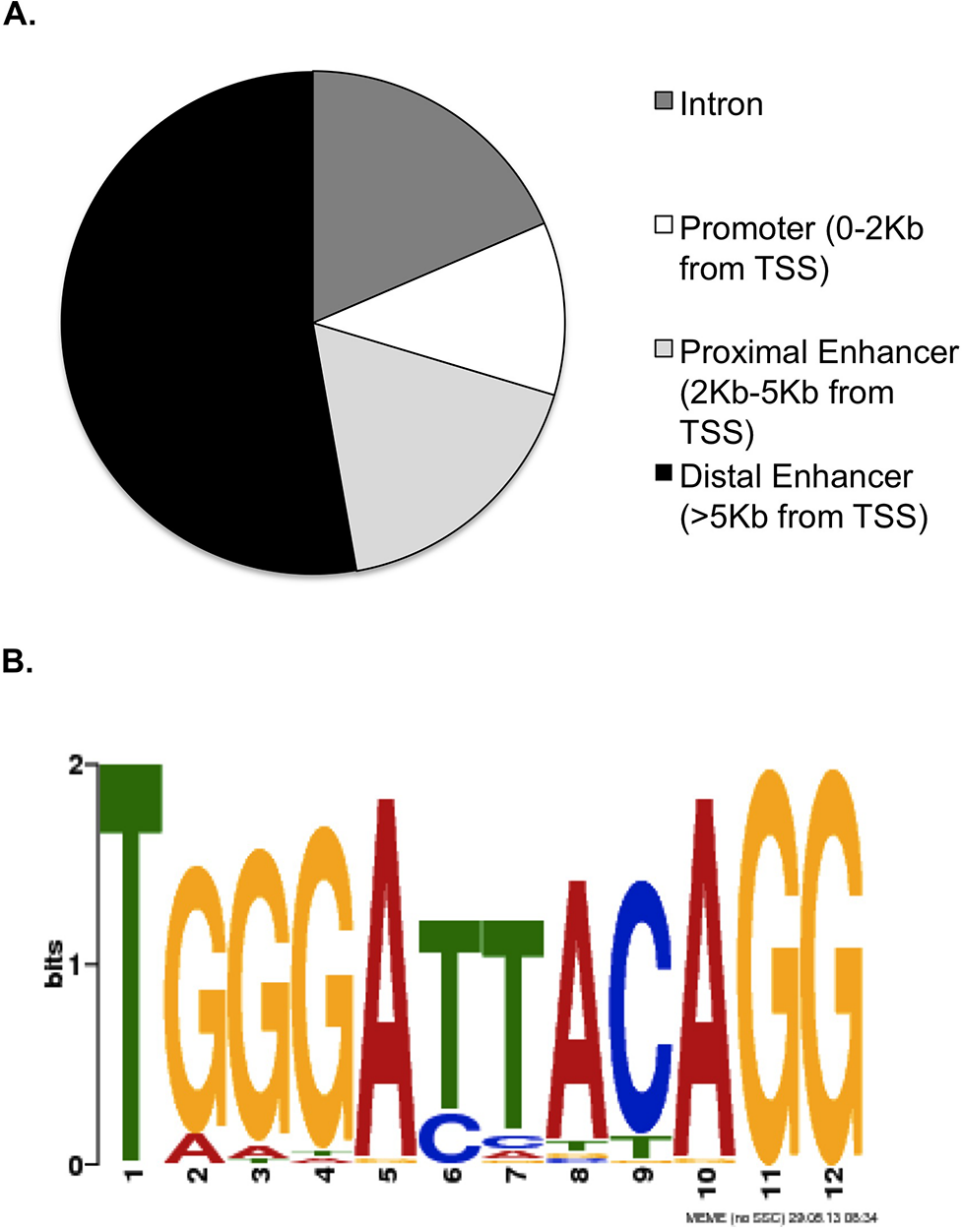
ARID3B binds to promoter and enhancer regions in a sequence-specific manner. (A) Distribution of ARID3B binding sites relative to the transcription start site of the nearest gene. (B) Graphical representation of the computationally-derived ARID3B consensus site, generated by MEME.

Next we assessed if the ARID3B bound genes cluster into distinct pathways or biological functions. The genomic regions surrounding each ARID3B binding site were scanned for Transcription Start Sites (TSS) in proximity, thus assigning each binding site to a likely regulatory region. Approximately 11% of the ARID3B binding sites located in this manner are in the immediate promoter region of a gene (defined as 0–2Kb from the TSS), 18% are in a nearby enhancer region (2–5Kb from the TSS), 52% are in more distant enhancer regions, and 19% are in introns (Fig 2).

Genes with nearby ARID3B binding were grouped by Gene Ontology (GO) terms of biological function (Table 1). Many of the putative ARID3B targets have GO terms associated with cell death and apoptosis (88 genes), heart development (21 genes), neuron development (47), and stem cell markers (6 genes). A smaller number of genes were found with GO terms for limb bud (2 genes) and facial-cranial formation (1 gene). We also noted that ARID3B binds to a number of genes involved in centromeres or metaphase (44 genes). These categories represent a subset of the biological functions that ARID3B may regulate and implicate ARID3B targets in many processes.

**Table 1:** Genes containing ARID3B binding sites, grouped by Gene Ontology.

Cell Death/Apoptosis	Heart Defects	Neuronal Defects	Limb Bud Formation	Metaphase	Stem Cell
ADORA1	ADAM19	ABL1	FGF8	AKAP9	APC
APC	ATM	AMIGO1	HOXD11	APC	MTF2
APP	CDKL1	ANK1		CCDC92	NOTCH2
ATL1	DSCR6	ANK3		CENPK	SOX21
ATM	FGF19	APP		CENPN	STAG2
BECN1	FGF8	ATL1		CEP152	ZCCHC11
BIRC3	FOXC1	CASR		CEP55	
BIRC7	FOXP1	CCK		CEP63	
BTC	GNAQ	CNTNAP2		CEP70	
CASP1	KDM6A	DRGX		DTL	
CD74	LEFTY1	EGFR		DYNLL1	
CDKN2A	MEF2D	FEZF2		DYNLL2	
COMP	MTERFD2	GRM7		FBXL7	
CXCL12	NFATC1	HCN1		FGFR1OP	
DAPK2	OVOL2	HRAS		HAUS4	
DPP6	RAF1	ITGAV		HAUS6	
EGFR	RBM20	ITGB1		HAUS7	
ERN1	RGS2	KAL1		ITGB3BP	
EYA1	RNF41	KLC1		KIF2A	
FABP1	T	LHX2		KIF3B	
FGF8	UTY	LMX1A		KIFAP3	
FIGNL1		MAF1		MDH1	
FOXC1		MAPT		NCAPD2	
FZD5		MATN2		NCAPG	
GCH1		MET		NEDD1	
GPX1		MYC		NIN	
GRIN1		MYH9		PAN3	
GRN		NKX2-1		PCGF5	
HGF		NPFF		PDE4DIP	
HRAS		NR4A3		PDS5B	
HSPA1A		OR10A4		PPP1R12A	
IL10		PAK2		PRKAR2B	
INS		PLXNC1		RAD21	
ITCH		PRKCQ		RGS14	
ITGAV		RAF1		RNF19A	
IVNS1ABP		RNF6		ROCK2	
JMY		ROCK2		SEH1L	
KIF1A		RPS6KA4		SORBS1	
LPAR1		SEMA3E		STAG2	
LRRK2		SEMA4F		SYTL4	
MITF		SEMA7A		TCHP	
MPO		SPTA1		WAPAL	
MTPAP		SPTAN1		WRN	
MYC		TAC1			
NAIP		TANC1			
NGF		TRPC7			
NOTCH2		WNT5A			
NR4A3					
PAK2					
PARK2					
PEA15					
PERP					
PLAC8					
PLAGL1					
PLP1					
POLB					
PRAMEF1					
PRAMEF11					
PRAMEF12					
PRAMEF15					
PRAMEF2					
PRAMEF22					
PRAMEF3					
PRAMEF4					
PRAMEF6					
PRAMEF7					
PRAMEF8					
PRAMEF9					
PROKR1					
PTGER3					
RAF1					
RIPK1					
RNF41					
SCXA					
SCXB					
SERPINB9					
SGMS1					
SIN3A					
SMAD6					
SPHK2					
STK4					
SYT14					
TFG					
TNFRSF19					
TP73					
TREX1					
WNT5A					
XRCC5					

ChIP-chip analysis identified 2367 genomic regions in bound by ARID3B in OVCA429 cells. ARID3B target genes were grouped by selected Gene Ontology (GO) terms for biological functions and the top genes from each group are represented.

### ARID3B regulates gene expression

We next wanted to identify ARID3B regulated genes that are involved in tumor growth we isolated cells from a mouse model of ovarian cancer. Skov3IP cells expressing red fluorescent protein (RFP) or 6XHis-ARID3B and RFP were injected into nude mice. Tumors were allowed to grow. We collected ascites or peritoneal washes from Skov3IP-6XHis-ARID3B or Skov3IP-RFP xenografts ascites as described [3]. RNA was collected from the malignant ascites form Skov3IP-ARID3B tumor bearing mice or peritoneal washes from mice with Skov3IP-RFP tumors. Microarray analysis was conducted using an Affymetrix Human Genome U133 Plus 2 GeneChip. In this experiment there were 813 genes with increased expression in response to ARID3B, and 201 genes with decreased expression (S2 Table). A more stringent calculation of "highly modified" genes (based on RMA normalization with False Discovery Rate less than 5%) yielded 132 genes with increased expression, and 39 with decreased expression. Similar to our ChIP-Chip data, these results were filtered by GO terms. Among the genes that are ARID3B induced are 44 cell death genes, 13 heart development genes, 17 neural development genes, 37 cell division genes, and 9 stem cell genes (Table 2). In agreement with our ChIP-ChIP data, several genes we identified as direct ARID3B targets were induced by ARID3B expression including NOTCH2, CASP1, CENPN, and CENPK. A statistical analysis of GO term distribution found that metaphase and centromere-associated terms were significantly over-represented among genes upregulated by ARID3B, while neuron development terms were over-represented among genes down-regulated by ARID3B (S3 Table).

**Table 2:** Genes significantly induced by ARID3B, grouped by Gene Ontology.

Cell Death/Apoptosis	Heart Defects	Neuronal Defects	Limb Bud Formation	Metaphase	Stem Cell
ANXA4	ADAM19	ABL2	GNAS	AURKA	LIF
ATG5	FOXL1	ANK1	MSX1	CCNB1	MED14
AXL	GLI3	ANK2	RUNX2	CENPJ	MED30
BARD1	HEXIM1	CA2	SOX9	CENPK	MSX1
BCLAF1	MSX1	CAP2		CENPN	NOTCH2
C9orf72	RBM20	COL6A3		CENPQ	RUNX2
CASP1	SALL1	GLI3		CEP55	SOX17
CD44	SHOX2	ITGA5		CEP78	TIAL1
CD70	SLC8A1	ITGB3		CETN3	WWTR1
CFLAR	SOD2	KIF4A		CKAP2	
CTGF	SOX17	LAMB1		CKAP2L	
CYR61	SOX9	PRSS12		CNTLN	
EGR3	SPARC	RGS10		HOOK3	
FAS		ROBO1		HYLS1	
GLI3		RPS6KA3		IST1	
HTATIP2		RPS6KA5		KIF15	
IFI6		TANC1		MARCKS	
IKBIP				MASTL	
ING2				MLF1IP	
ITGA6				NFU1	
IVNS1ABP				NSL1	
LPAR1				NUF2	
MSX1				PLK4	
NFKB1				POC5	
NFKBIA				PPP1CC	
NOTCH2				RGCC	
PARP4				SDCCAG8	
PHLDA1				SORBS1	
PMAIP1				SPC25	
RPS6KA3				SPICE1	
SCG2				TNFAIP3	
SERPINB9				TUBGCP3	
SFRP1				TXNDC9	
SMAD3				WDR67	
SNAI2				XPO1	
SOD2				ZWILCH	
SOX9				ZWINT	
STK17A					
STK17B					
TARDBP					
TIAL1					
TNFAIP3					
TNFRSF10A					
TRAF3					

Using microarray we identified ARID3B induced genes and classified them based on highly represented Gene Ontology (GO) terms for biological functions. The top genes from each group are represented.

### Characterizing the ARID3B binding site

**Fig 3:**
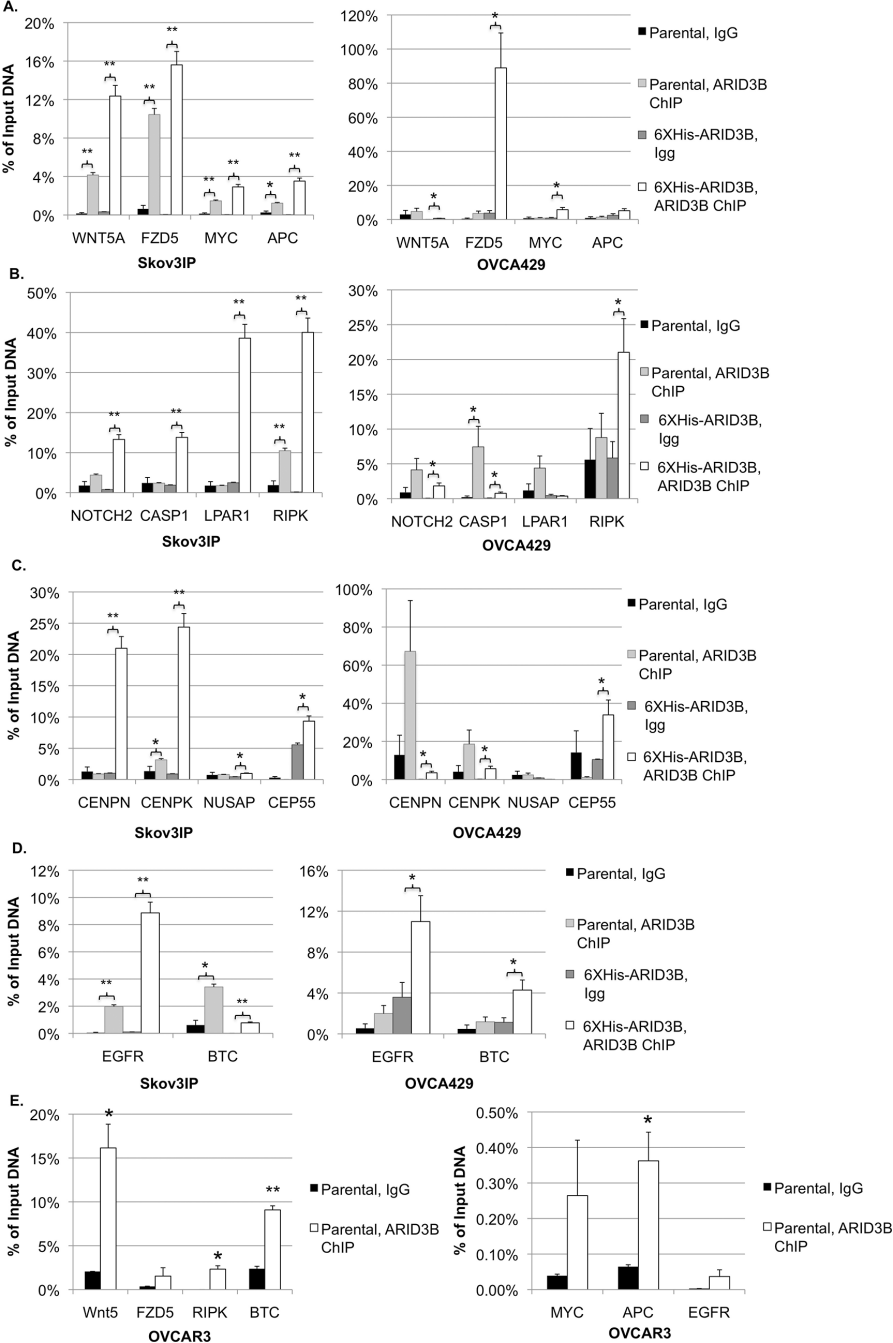
ARID3B binds gene regulatory regions for genes in Wnt Signaling, Cell Death, Cell Division, and EGFR signaling. Chromatin Immunoprecipitation (ChIP) followed by qPCR was performed on parental and 6XHis-ARID3B Skov3IP and OVCA429 cells to detect binding of ARID3B to target genes in key pathways. All numbers are reported as relative binding compared to the corresponding input DNA sample. Brackets indicate that binding in ARID3B-ChIP samples is significantly higher than the corresponding background (IgG) sample (*—p-value < 0.05, **—p-value < 0.005). N = 3. We validated ARID3B binding to genes with Gene Ontology (GO) terms relating to (A) Wnt Signaling, (B) Cell Death and Apoptosis, or (C) Cell Division, or (D) EGFR signaling. (E) Similar ChIP results in high-grade serous ovarian cancer cell line OVCAR3.

The consensus binding site of ARID3B's paralogue ARID3A was previously shown to be "AATTAA" [5]. As described above, genomic sequences bound by ARID3B were obtained by ChIP-Chip. These sequences were analyzed using two separate motif-finding algorithms (MEME [32] and ALLEGRO [34]) to find a common AT-rich motif. Both methods yielded a consensus binding motif of "TGGGATTACAG." (Fig 2) To demonstrate the functionality of this computationally derived motif, we performed ChIP to detect ARID3B binding to the regulator regions of genes identified via ChIP-Chip. Binding to the regulatory regions of these genes was determined using qPCR primers designed to amplify a 200–300bp region containing one or more putative ARID3B binding sites that we identified bioinformatically. ChIP samples were prepared from Skov3IP and OVCA429 ovarian cancer cells, using both parental lines and cell lines expressing 6XHis-ARID3B (Fig 1). Target genes were selected for validation based on the ChIP-Chip data, and the biological relevance of the gene. The selected target genes were divided into 4 categories of Gene Ontology: Wnt signaling (Wnt5A, FZD5, MYC, APC) (Fig 3A), Cell death-associated (NOTCH2, CASP1, LPAR1, and RIPK) (Fig 3B), Cell division (CENPN, CENPK, NUSAP, and CEP55) (Fig 3C), and EGFR signaling (EGFR and BTC) (Fig 3D). For each region, the qPCR results of ARID3B ChIP were normalized to an input DNA sample, and compared to a negative (IgG) control. As shown in Fig 3, nearly all selected target regions showed ARID3B binding substantially above the negative control in at least one cell line. Relative binding in ARID3B ChIP versus the equivalent negative control was generally much higher in cells overexpressing ARID3B, in comparison to the parental cell lines (Fig 3). Similar results were found when conducting ChIP on high-grade serous ovarian cancer cells (OVCAR3), with significant ARID3B binding found to Wnt5a, RIPK, BTC, and APC (Fig 3E).

### ARID3B alters the expression of genes in key cellular pathways

**Fig 4:**
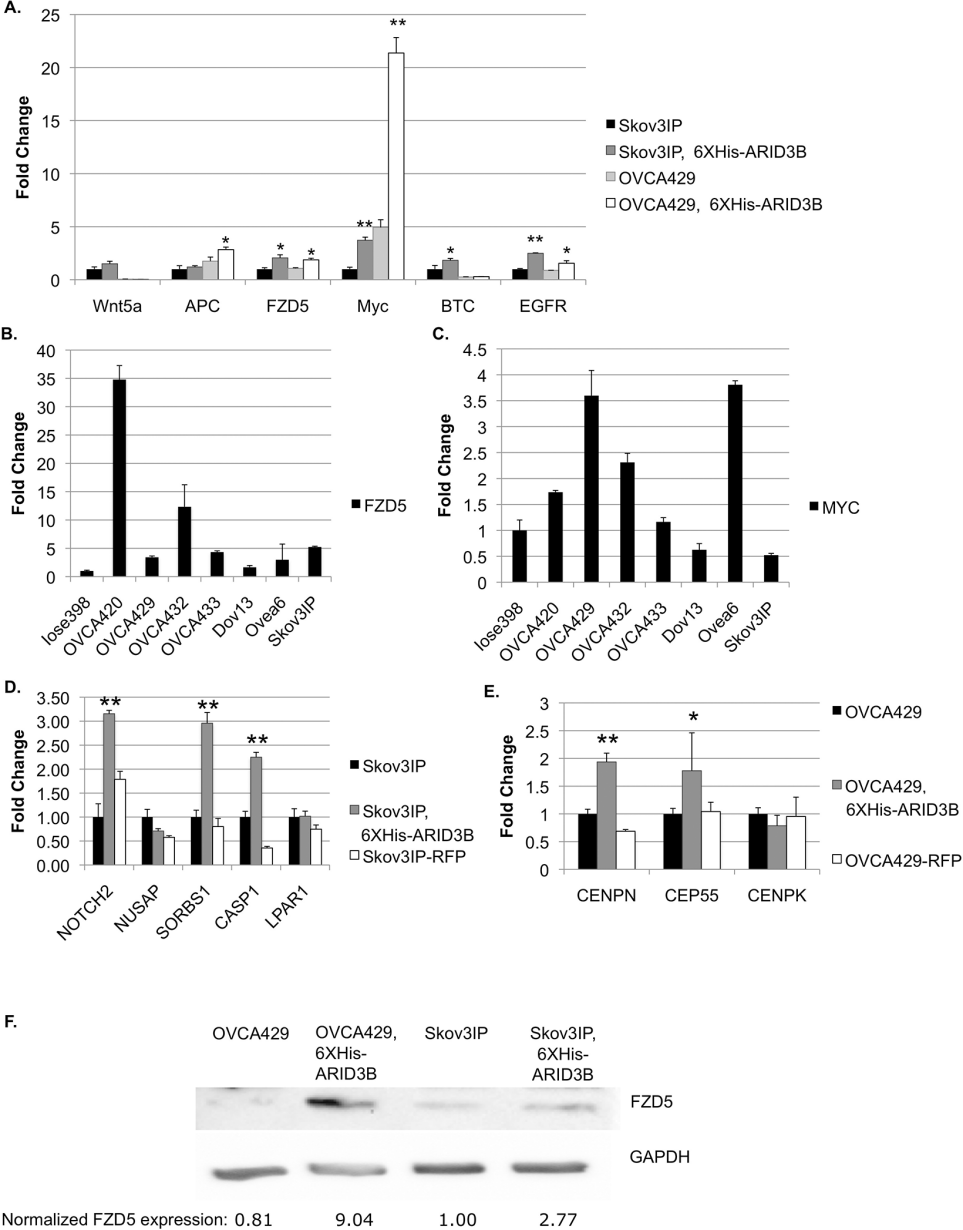
ARID3B induces expression of target genes. (A) qRT-PCR was performed for target genes on OVCA429 and Skov3IP parental cells and OVCA429 and Skov3IP cells expressing 6xHis-ARID3B. N = 5 (B and C) qRT-PCR was performed on total RNA prepared from IOSE398 and a panel of ovarian cancer cell lines to measure the expression of putative ARID3B target genes FZD5 and MYC. N = 3 (D) qRT-PCR analysis on Skov3IP parental cells, Skov3IP-6XHis-ARID3B, and Skov3IP-RFP cells. N = 3 (E) qRT-PCR analysis on OVCA429 parental cells, 6XHis-ARID3B, and RFP-expressing OVCA429 cells. N = 4. * = p< 0.05, ** = p< 0.005 (F) Western Blot using lysates OVCA429, OVCA-6xHis-ARID3B, Skov3 and Skov3-6xHis-ARID3B cells for FZD5. N = 2.

Next we ascertained if ARID3B expression alters expression of putative target genes. Exogenous expression of ARID3B increased genes in the Wnt and EGF signaling pathways by qRT-PCR. In OVCA429 cells, APC, FZD5, MYC, and EGFR are induced by ARID3B. In Skov3IP cells, ARID3B increases FZD5, MYC, BTC, and EGFR (Fig 4A). The consistent induction of FZD5 and MYC is especially interesting, considering that both are frequently expressed at high levels in ovarian cancer cells compared to immortalized ovarian surface epithelial cells (IOSE398) (Fig 4B and 4C). Additionally, we confirmed that ARID3B induces the expression of many predicted targets: ARID3B upregulated NOTCH2, SORBS1, and CASP1 in Skov3IP cell lines (Fig 4D). These genes were considered of interest due to their Gene Ontology terms. ARID3B also binds several metaphase and centromere-associated genes (Table 1). We confirmed the binding of ARID3B to regulatory regions in CEP55, CENPN, and CENPK using ChIP (Fig 3C). In OVCA429 cells, CEP55 and CENPN are significantly upregulated by ARID3B (Fig 4E).

Since Wnt signaling is implicated in many type of tumors including ovarian cancer we further investigated regulation of FZD5 by ARID3B. Upregulation of FZD5 was further confirmed by western blot (Fig 4F) in which protein expression of FZD5 increased 9-fold in OVCA429 cells and 2.8-fold in Skov3 cells in response to expression of 6xHis-ARID3B. This demonstrates that ARID3B regulates FZD5 *in vitro*.

**Fig 5:**
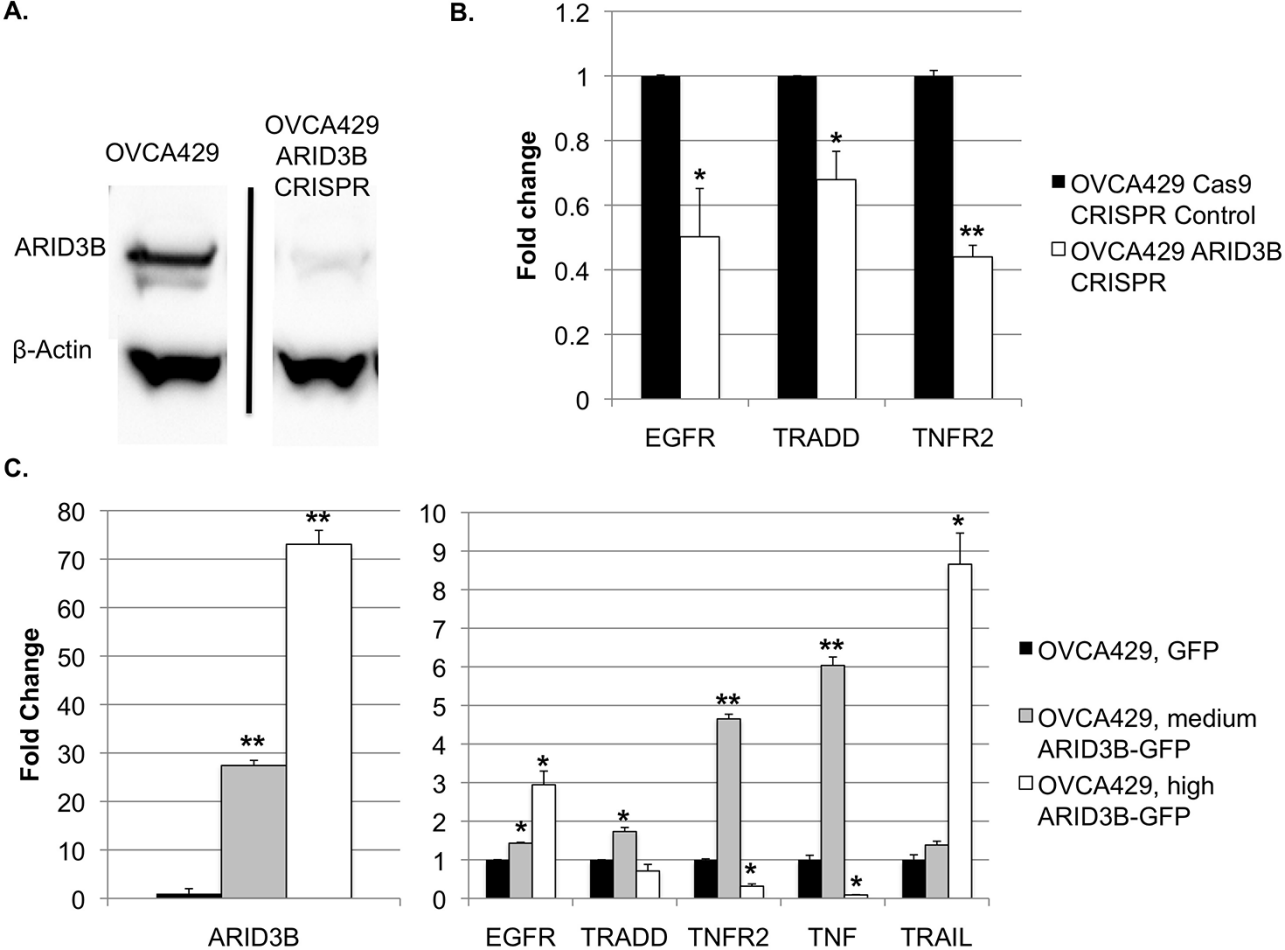
CRISPR-induced editing of ARID3B causes loss of ARID3B, and downregulation of ARID3B targets. (A) Western blot using whole-cell lysate from OVCA429 and OVCA429 ARID3B CRISPR cells, with detection for ARID3B. N = 2. (B) qRT-PCR was performed for EGFR, TRADD, and TNFR2 on total RNA from OVCA429 cells transduced with Cas9 nuclease and a control scrambled CRISPR guide RNA, and OVCA429 cells with ARID3B edited by targeted CRISPR vectors. (C) qRT-PCR was performed for ARID3B on OVCA429-GFP and OVCA429 cells sorted for medium ARID3B-GFP or high ARID3B-GFP expression. (D) qRT-PCR was performed for EGFR, TRADD, TNFR2, TNF, and TRAIL on OVCA429-GFP, OVCA429 medium ARID3B-GFP, and OVCA429 high ARID3B-GFP cells. N = 3. * = p<0.05. ** = p<0.005.

To further support the role of ARID3B in regulating gene expression, OVCA429 cells were transfected with vectors expressing Cas9 nuclease and CRISPR guide RNAs targeting ARID3B. Significant loss of ARID3B expression was confirmed by western blot (Fig 5A) and frameshift mutations were verified by sequencing (data not shown). Expression of ARID3B target genes was measured using qPCR as before, comparing OVCA429 cells with CRISPR-edited ARID3B against OVCA429 cells containing a control Cas9 and scrambled sgRNA vector. We found significantly decreased expression of EGFR in the CRISPR-edited cells (Fig 5B), and the same for pro-apoptotic targets TRADD and the TNF receptor TNFR2, which our lab had previously verified to be upregulated by ARID3B[23].

To assess if concentration of ARID3B impacts gene regulation, we transduced a OVCA429 cells with a lentiviral ARID3B fused to green fluorescent protein (GFP) (ARID3B-GFP), cells were sorted by fluorescent activated cell sorting (FACS) for high (73-fold increase over endogenous ARID3B) or moderate (27-fold increase over endogenous ARID3B) levels of ARID3B-GFP. Moderate expression of ARID3B induced EGFR, TRADD, TNFR2, and TNF (Fig 5C). High levels of exogenous ARID3B resulted in lower expression TNF and TNFR2, but induced expression of EGFR and TRAIL. These data suggest that the concentration of ARID3B in a particular cell or cell type results in differential target gene expression.

### ARID3B and Frizzled Receptor 5 increase Wnt signaling and adhesion to Extracellular Matrix components

**Fig 6:**
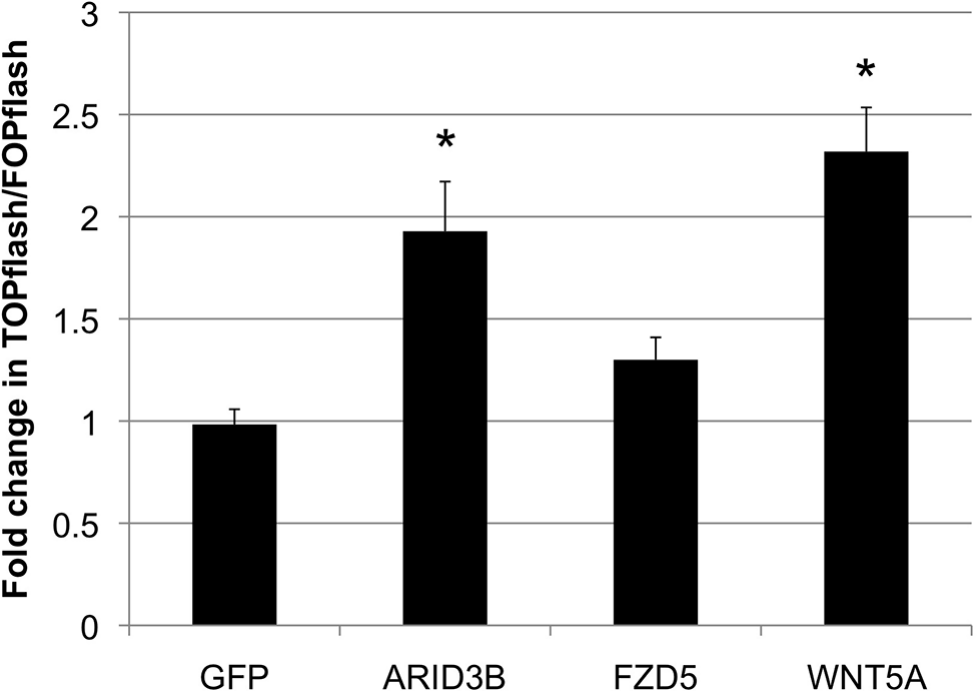
ARID3B and FZD5 increase β-catenin-dependent transcription in 293FT cells. (A) TOPflash reporter activity in 293FT cells transfected with either TOPflash or FOPflash along with vectors expressing ARID3B, FZD5, or Wnt5a. FOPflash reporter plasmids containing mutated TCF binding sites were used for normalization. Conditions were run in triplicate. N = 3. * = p< 0.05.

Since ARID3B regulates the expression of Wnt signaling pathway genes and elevated Wnt signaling is associated with ovarian cancer progression, we examined this relationship. A TOPflash assay was conducted to measure β-catenin-dependent (TCF/LEF) transcription in cells co-transfected with vectors expressing ARID3B (pLenti-suCMV-Rsv), FZD5 (pLenti-C-mGFP) or Wnt5a (pLenti-C-mGFP). Due to poor transfection efficiency in our ovarian cancer cell lines, this experiment was conducted in 293FT cells. It should be noted that in all of our experiments using ovarian cancer cell lines (OVCA429, Skov3IP, Skov3, and OVCAR3) it was necessary to use lentiviral transduction to introduce 6xHis-ARID3B, RFP, or FZD5 into the cells. We were unable to transfect the cells with the TOPflash/FOPflash plasmids. However, we found that in 293FT cells TCF/LEF activation is significantly higher in cells transfected with ARID3B or Wnt5a (Fig 6A) suggesting that ARID3B activates Wnt signaling in 293FT cells. However, a similar experiment using 3T3 "Leading Light" Cells did not show any statistically-significant difference when the cells were transfected with ARID3B or Wnt5a (data not shown). Therefore at this time we cannot conclude if induction of FZD5 by ARID3B results in activation of TCF/LEF induced transcription.

**Fig 7:**
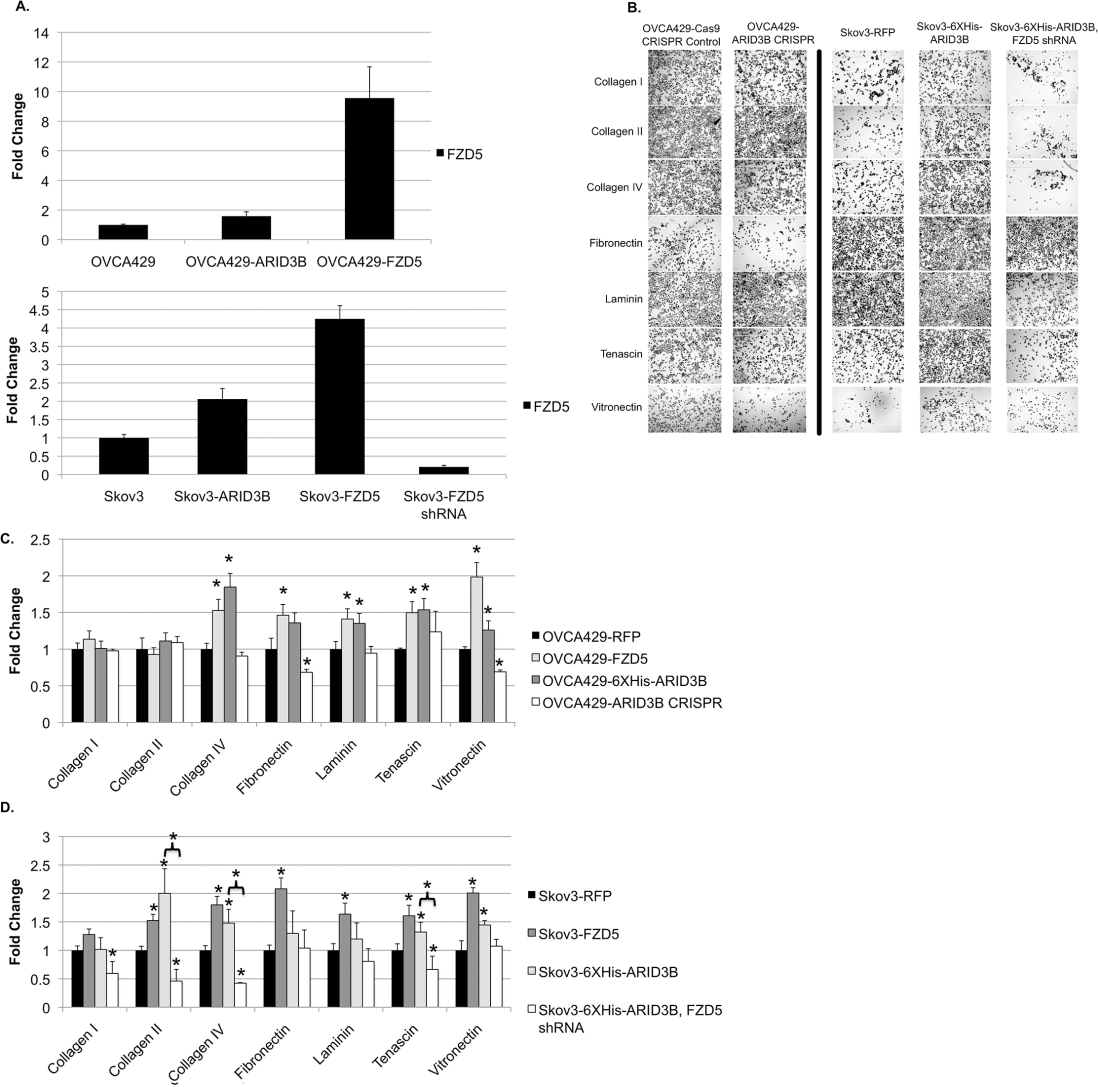
ARID3B and FZD5 increases cell adhesion to extracellular matrices. (A) qRT-PCR was used to measure FZD5 expression in OVCA429 and Skov3 cell lines transduced to over-express FZD5, or knock it down with shRNA. (B) (B) Representative images of ECM adhesion assays in OVCA429 and Skov3, 10X original magnification. (C and D) ECM adhesion assays were performed on OVCA429 (C) and Skov3 (D) cells, 6xHis-ARID3B expressing cells (dark gray bars) and FZD5 transduced cells (light grey bars). OVCA429 cells with ARID3B edited by CRISPR (white bars) are also included. Skov3 cells with elevated ARID3B combined with FZD5 shRNA (white bars) are compared to Skov3 6xHis-ARID3B cells. Data was normalized to Skov3-RFP cells. Statistical comparisons for C and D are relative to the RFP control cell line, unless otherwise indicated. N = 3. * = p< 0.05.

We previously found that ARID3B alters actin cytoskeleton and adhesion [3]. To assess if ARID3B modulates changes in adhesion potentially through Wnt signaling, we tested the effects of ARID3B or FZD5 expression (Fig 7A) in ovarian cancer cells on adhesion to ECM components (Fig 7B, 7C, and 7D). OVCA429 and Skov3 expressing ARID3B or FZD5 were plated onto a Millipore Colorimetric ECM Cell Adhesion Array Kit and compared to OVCA429 and Skov3 control cells. In addition, we compared Skov3 6x-His-ARID3B, but also transduced with an shRNA for FZD5. OVCA429 cells (Fig 7C) overexpressing FZD5 displayed increased adhesion to collagen IV (1.53 fold, p = 0.010), fibronectin (1.36 fold, p = 0.035), laminin (1.41 fold, p = 0.029), tenascin (1.50 fold, p = 0.006), and vitronectin (1.98 fold, p = 0.001). When ARID3B was edited using CRISPR technology, fibronectin (0.68 fold, p = 0.03) and vitronectin (0.69 fold, p = 0.002) adhesion was decreased compared to adhesion in 6x-His-ARID3B expressing cells. In Fig 7D, Skov3 cells expressing FZD5 showed similar increases in adhesion to ECM: collagen II (1.66 fold, p = 0.029), collagen IV (1.96 fold, p = 0.019), fibronectin (2.27 fold, p = 0.014), laminin (1.79 fold, p = 0.023), tenascin (1.75 fold, p = 0.024), and vitronectin (2.19 fold, p = 0.015). Cells expressing 6x-His-ARID3B displayed similarly increases in ECM adhesion (Fig 7C & 7D). When FZD5 was knocked down using shRNA, Skov3 cells over-expressing ARID3B exhibited decreased adhesion to collagen I, collagen II (p = 0.012), collagen IV (p = 0.008), and tenascin (p = 0.042). Most importantly, FZD5 shRNA prevented the increase in adhesion to collagen II, collagen IV, and tenascin that that results form expression of 6x-His-ARID3B. Collectively, this data suggests ARID3B induces increased in adhesion by regulation of its target gene FZD5.

## Discussion

Previous work identified the DNA binding protein ARID3B as a critical regulator of embryonic development and tumor growth [3, 10, 37]. However, how ARID3B regulates these processes is poorly understood. To begin to shed light on the molecular mechanisms by which ARID3B regulates ovarian cancer progression, we identified direct targets of ARID3B. We demonstrate that ARID3B binds regulatory regions of target genes in a sequence specific fashion and alters the expression of endogenous target genes in ovarian cancer cell lines.

We have demonstrated a novel binding site, determined computationally from a collection of hundreds of DNA sequences bound by ARID3B through ChIP-Chip analysis. Our calculated ARID3B site is both a variation as well as an expansion on the previously-known ARID3A site, which has allowed for very strong predictions using motif-matching algorithms such as POSSUM (http://zlab.bu.edu/~mfrith/possum/). There are some limitations to our methods, however, in that our ChIP-chip data is restricted to sites available on our promoter array, as opposed to a genome-wide ChIP-seq approach used recently to locate ARID3A binding sites[7]. It is notable that in recent ChIP-seq studies, ARID3A and Oct4 bind to a consensus site of "ATGCAAAT", notably different from the previously-established site of "(G/A)ATTAA". A recent study found that ARID3B binds to a region of the Oct4 promoter containing the sequence "AATAAAAATAA"[9]. We did not find this sequence to be over-represented among ARID3B-bound regions in our ChIP experiments, though we did find matches for our putative ARID3B binding site in the Oct4 promoter. These discrepancies may be the result of differing ARID3B binding partners, or differences between our ovarian cancer cell lines, and the stem cells used in other studies.

**Fig 8:**
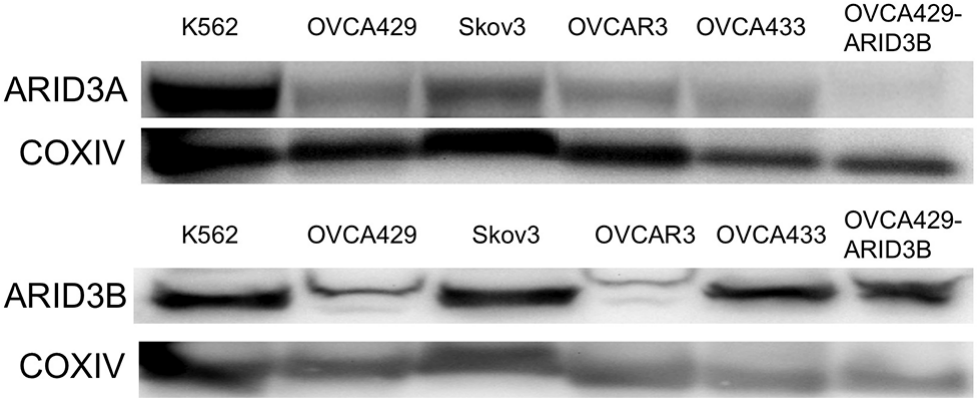
ARID3A and ARID3B protein expression levels in ovarian cancer cells. Western blot on K562 (positive control for ARID3A chronic myelogenous leukemia derived), OVCA429, Skov3, OVCAR3, OVCA433, and OVCA429-ARID3B cells (positive control for ARID3B expression). Western blot was performed for protein expression of ARID3A, and ARID3B. COXIV is used as a loading control.

It was published that exogenous ARID3B forms a heterodimer with ARID3A in COS-7 cells [38]. However, the knockout phenotypes for ARID3A and ARID3B are distinct and not overlapping [13]. *Arid3b* ^*-/-*^ embryos die earlier with neural crest defects while *Arid3a* ^*-/-*^ embryos exhibit hematopoietic defects. Additionally, ARID3A and ARID3B have only partially overlapping patterns of expression in adult tissues. While both paralogues are expressed at high levels in lung and spleen [37], they exhibit opposite patterns of expression in kidney and stomach tissues. ARID3B is increased in kidney tumors compared to normal kidney while ARID3A decreases. Conversely, ARID3A is increased in stomach tumors compared to normal stomach; ARID3B decreases during stomach tumor progression [37]. Given that their binding motifs are similar, we wanted to assess if ARID3A is co-expressed in our cell lines. In Fig 8, we demonstrate that ARID3A is expressed at varying levels in our ovarian cancer cell lines suggesting that heterodimers may form. Further studies will identify the ARID3B transcriptional complex.

In a ChIP-Chip experiment, over 2,000 genomic regions were bound by ARID3B (S1 Table). Microarray determined that overexpression of ARID3B upregulated 813 genes and repressed 201 genes (S2 Table). Further work is needed to determine how ARID3B directly activates and/or represses gene expression. Gene regulation by ARID3B varied by cell type, in that some target genes were regulated in one cell line but not another. This variable regulation suggests that target gene activation likely requires other factors, which may be expressed at different levels in a cell-line dependent manner. In addition, we noticed that the endogenous expression of ARID3B targets displayed significant variation between cell lines. ARID3B induced NOTCH2 expression in Skov3IP cells, in which endogenous levels of NOTCH2 are comparatively low. ARID3B did not increase NOTCH2 expression in OVCA429 cells, in which NOTCH2 is already expressed at very high levels. This notion of context-specific gene regulation may explain the role for ARID3B in different tissues[37].

The specific gene targets we discovered provide insight into how ARID3B acts during development and in cancer. *Arid3b* null embryos have several abnormalities, including heart defects, neurological defects, and retarded limb bud and facial-cranial development [11–13]. Additionally, ARID3B induces TNF induced death [23] and promotes neuroblastoma [14] and ovarian tumor growth [33]. In agreement with these studies, our data demonstrate that many direct ARID3B targets have GO terms related to cell death, neural development, and heart defects. Additionally, many pathways involved in both development and cancer are regulated by ARID3B. This includes genes in the Wnt, EGFR, TNF, and NOTCH signaling pathways. A Chi-squared test revealed GO terms that are significantly over-represented among these ARID3B-regulated genes. Several of these over-represented GO terms relate to metaphase and centromeres, and we confirmed that CENPN, CENPK, and CEP55 are direct targets of ARID3B. CEP55 overexpression increases tumorgenicity in gastric carcinoma [39], while high expression of CENP-A correlates with poor survival in epithelial ovarian cancer [40]. Therefore, many of the target genes that are ARID3B-regulated are implicated in cancer.

ARID3B induction of the Wnt signaling pathway is particularly interesting. ARID3B activates four Wnt-pathway genes: WNT5A, FZD5, APC, and MYC. Wnt signaling enhances tumor cell proliferation and tumor development, and FZD5 expression correlates with poor prognosis in ovarian cancer [16–20, 41]. We showed that FZD5 is overexpressed in ovarian cancer cell lines compared to IOSE398 cells; this finding agrees with studies of ovarian cancer which find that FZD5 expression correlates with advanced malignancy [42]. In light of this, it is notable that FZD5 is increased by overexpression of ARID3B. TCF/LEF induced transcriptional activity is induced by ARID3B transfected 293FT cells, but since we were unable to perform TOPFlash assays in our ovarian cancer cells we do not know if ARID3B activation of FZD5 results in TCF/LEF mediated gene expression.

Upregulation of either ARID3B or FZD5 increased adhesion to several ECM components, particularly vitronectin, fibronectin, and collagen IV. This is consistent with data showing that Wnt signaling triggered through FZD5 with the WNT7A ligand increases adhesion[20]. We provide further support for the role of FZD5 in adhesion with an FZD5 shRNA knockdown, which reduces binding to multiple forms of collagen (Fig 7). Notably, this is the first demonstration that FZD5 is critical for adhesion to collagen. Since adhesion is important for ovarian peritoneal metastasis [43, 44], ARID3B activation of Wnt signaling components may promote metastasis by enhancing cell matrix adhesion.

Finally, ARID3B binds an enhancer region of EGFR and the EGFR ligand BTC increasing their expression. We previously demonstrated that ARID3B is induced by EGFR signaling [2, 23]. We now demonstrate that ARID3B directly activates EGFR, demonstrating a feed-forward pathway where EGFR signaling through ARID3B leads to increased EGFR expression. EGFR is often overexpressed in ovarian cancer and correlates with poor prognosis [45–50]. We surmise that ARID3B regulation of EGFR may be a mechanism to maintain elevated EGFR expression [47]. This conclusion is further supported by the decrease in EGFR expression observed when ARID3B is edited using CRISPR technology. The use of CRISPR to reduce functional ARID3B is key to our work going forward, as it will clarify whether ARID3B is essential for regulation of specific target genes.

Our data demonstrate that ARID3B regulates a number of tumor promoting pathways and ARID3B overexpression leads to increased tumor growth and metastasis [33]. However, the role of ARID3B in tumor development is complicated, due to the large number of targets, and seemingly contradictory roles of ARID3B in cellular behavior. As noted before, ARID3B increases tumor growth[33] and activates cell death pathways[23]. ARID3B promotes tumor growth in neuroblastoma and ovarian cancer, but ARID3B expression decreases with progression of esophageal and stomach cancer [14, 37]. This suggests that ARID3B’s role in cancer may be context dependent. We demonstrate (Fig 5) that the differential regulation of pathways is at least in part due to the expression levels of ARID3B. ARID3B gene editing decreases expression of EGFR, TRADD, and TNFR2, while moderate over-expression (27-fold) of ARID3B increases all of these targets. However, a higher level of ARID3B over-expression (73-fold) decreases the expression of TRADD and TNFR2. Future experiments will dissect the mechanism for how ARID3B concentration dictates target gene expression and how regulation of these pathways contributes to ovarian tumor growth and metastasis.

In conclusion, we identified direct targets of ARID3B, which include members of the Wnt, EGF, NOTCH, and signaling pathways. In particular, ARID3B induction of the Wnt receptor FZD5 is important in increasing tumor cell adhesion, which may contribute to metastasis.

## Supporting Information

S1 Fig ARID3B expression in OVCA429 and Skov3IP. Uncropped western blot showing detection of ARID3B in parental OVCA429 and Skov3IP cell lines compared to the cell lines transduced with 6XHis-ARID3B, as shown in Fig 1. Uncropped blot for β-Actin loading controls is also included.
(TIF)

S2 Fig FZD5 expression in OVCA429 and Skov3IP. Uncropped western blot showing detection of FZD5 in parental OVCA429 and Skov3IP cell lines compared to cell lines transduced with pGMP-FZD5, as shown in Fig 4. Uncropped blot for GAPDH loading controls.
(TIF)

S3 Fig ARID3B expression in parental OVCA429 and CRISPR-edited OVCA429. Uncropped western blot for ARID3B in OVCA429 parental cells, versus OVCA429 cells edited with a CRISPR sgRNA targeting ARID3B, as shown in Fig 5. Samples loaded in other lanes were not used in subsequent experiments. Uncropped blot for β-Actin loading controls.
(TIF)

S4 Fig ARID3B and ARID3A expression in cell lines. Uncropped western blot for ARID3B and ARID3A in various ovarian cancer cell lines, as shown in Fig 7. Uncropped blot for COXIV loading control.
(TIF)

S1 Table ARID3B ChIP-on-chip summary data. Listing of genomic regions with significant binding to the Nimblegen Human 2.1M Deluxe Promoter Array, following collection of DNA fragments bound by ARID3B. Significant differences are determined by MAT score. Nearest genes are listed under "Annotation".
(XLSX)

S2 Table Changes in gene expression in 6XHis-ARID3B cell lines, measured by microarray. Significant changes in gene expression are listed, based on data collected using an Affymetrix Human Genome U133 Plus 2 GeneChip.
(XLSX)

S3 Table Over-represented Gene Ontology terms among genes upregulated or downregulated by ARID3B. Each gene identified to have significantly altered expression in cells over-expressing ARID3B (determined by microarray) was sorted by associated Gene Ontology (GO) terms. The frequency of GO terms was compared against their corresponding frequencies across the entire genome, and over-represented terms were calculated using a Chi-Squared test.
(XLSX)

S4 Table Letter-probability Matrix describing the putative ARID3B binding site. Based on an alignment of many sequences bound by ARID3B, the following binding site was computed, with probabilities assigned to each base at each position.
(XLSX)

S5 Table Location of ARID3B binding sites confirmed by ChIP. For each ARID3B binding site validated by ChIP and subsequent qPCR (Fig 3), the distance to the transcription start site is listed, along with a description of the binding site's location.
(XLSX)

## References

[1] American Cancer Society (2014) Cancer Facts and Figures.

[2] Cowden Dahl KD, Dahl R, Kruichak JN, Hudson LG. The epidermal growth factor receptor responsive miR-125a represses mesenchymal morphology in ovarian cancer cells. Neoplasia. 2009;11(11):1208–15. Epub 2009/11/03. 19881956 10.1593/neo.09942 PMC2767222

[3] Roy L, Samyesudhas SJ, Carrasco M, Li J, Dahl R, Cowden Dahl KD. ARID3B accererates ovarian cancer progression in vivo by inducing multiple tumor promoting pathways. Oncotarget. 2014;In Press.

[4] Gregory SL, Kortschak RD, Kalionis B, Saint R. Characterization of the dead ringer gene identifies a novel, highly conserved family of sequence-specific DNA-binding proteins. Mol Cell Biol. 1996;16(3):792–9. Epub 1996/03/01. 8622680 10.1128/mcb.16.3.792 PMC231059

[5] Herrscher RF, Kaplan MH, Lelsz DL, Das C, Scheuermann R, Tucker PW. The immunoglobulin heavy-chain matrix-associating regions are bound by Bright: a B cell-specific trans-activator that describes a new DNA-binding protein family. Genes Dev. 1995;9(24):3067–82. Epub 1995/12/15. . 8543152 10.1101/gad.9.24.3067

[6] Raney BJ, Cline MS, Rosenbloom KR, Dreszer TR, Learned K, Barber GP, et al ENCODE whole-genome data in the UCSC genome browser (2011 update). Nucleic Acids Res. 2011;39(Database issue):D871–5. Epub 2010/11/03. 10.1093/nar/gkq1017gkq1017 [pii]. 21037257 PMC3013645

[7] Rhee C, Lee BK, Beck S, Anjum A, Cook KR, Popowski M, et al Arid3a is essential to execution of the first cell fate decision via direct embryonic and extraembryonic transcriptional regulation. Genes Dev. 2014;28(20):2219–32. Epub 2014/10/17. 10.1101/gad.247163.114 28/20/2219 [pii]. 25319825 PMC4201284

[8] Numata S, Claudio PP, Dean C, Giordano A, Croce CM. Bdp, a new member of a family of DNA-binding proteins, associates with the retinoblastoma gene product. Cancer Res. 1999;59(15):3741–7. Epub 1999/08/14. . 10446990

[9] Chien CS, Wang ML, Chu PY, Chang YL, Liu WH, Yu CC, et al. Lin28B/Let-7 Regulates Expression of Oct4 and Sox2 and Reprograms Oral Squamous Cell Carcinoma Cells to a Stem-like State. Cancer Res. 2015. Epub 2015/04/11. doi: canres.2215.2014 [pii] 0008-5472.CAN-14-2215 [pii] 10.1158/0008-5472.CAN-14-2215 . 25858147

[10] Wilsker D, Patsialou A, Dallas PB, Moran E. ARID proteins: a diverse family of DNA binding proteins implicated in the control of cell growth, differentiation, and development. Cell Growth Differ. 2002;13(3):95–106. Epub 2002/04/18. . 11959810

[11] Casanova JC, Uribe V, Badia-Careaga C, Giovinazzo G, Torres M, Sanz-Ezquerro JJ. Apical ectodermal ridge morphogenesis in limb development is controlled by Arid3b-mediated regulation of cell movements. Development. 2011;138(6):1195–205. Epub 2011/02/11. 10.1242/dev.057570 dev.057570 [pii]. . 21307092

[12] Takebe A, Era T, Okada M, Martin Jakt L, Kuroda Y, Nishikawa S. Microarray analysis of PDGFR alpha+ populations in ES cell differentiation culture identifies genes involved in differentiation of mesoderm and mesenchyme including ARID3b that is essential for development of embryonic mesenchymal cells. Dev Biol. 2006;293(1):25–37. Epub 2006/03/15. doi: S0012-1606(05)00889-4 [pii] 10.1016/j.ydbio.2005.12.016 . 16530748

[13] Webb CF, Bryant J, Popowski M, Allred L, Kim D, Harriss J, et al The ARID family transcription factor bright is required for both hematopoietic stem cell and B lineage development. Mol Cell Biol. 2011;31(5):1041–53. Epub 2011/01/05. 10.1128/MCB.01448-10 MCB.01448-10 [pii]. 21199920 PMC3067827

[14] Kobayashi K, Era T, Takebe A, Jakt LM, Nishikawa S. ARID3B induces malignant transformation of mouse embryonic fibroblasts and is strongly associated with malignant neuroblastoma. Cancer Res. 2006;66(17):8331–6. Epub 2006/09/05. doi: 66/17/8331 [pii] 10.1158/0008-5472.CAN-06-0756 . 16951138

[15] Kobayashi K, Jakt LM, Nishikawa SI. Epigenetic regulation of the neuroblastoma genes, Arid3b and Mycn. Oncogene. 2013;32(21):2640–8. Epub 2012/07/04. 10.1038/onc.2012.285 onc2012285 [pii]. 22751132 PMC3664305

[16] Arend RC, Londono-Joshi AI, Straughn JM Jr., Buchsbaum DJ. The Wnt/beta-catenin pathway in ovarian cancer: a review. Gynecol Oncol. 2013;131(3):772–9. Epub 2013/10/16. 10.1016/j.ygyno.2013.09.034 S0090-8258(13)01252-3 [pii]. . 24125749

[17] Arend RC, Londono-Joshi AI, Samant RS, Li Y, Conner M, Hidalgo B, et al Inhibition of Wnt/beta-catenin pathway by niclosamide: A therapeutic target for ovarian cancer. Gynecol Oncol. 2014;134(1):112–20. Epub 2014/04/17. 10.1016/j.ygyno.2014.04.005 S0090-8258(14)00862-2 [pii]. . 24736023

[18] Peng C, Zhang X, Yu H, Wu D, Zheng J. Wnt5a as a predictor in poor clinical outcome of patients and a mediator in chemoresistance of ovarian cancer. Int J Gynecol Cancer. 2011;21(2):280–8. Epub 2011/01/29. 10.1097/IGC.0b013e31820aaadb 00009577-201102000-00014 [pii]. . 21270611

[19] Ford CE, Punnia-Moorthy G, Henry CE, Llamosas E, Nixdorf S, Olivier J, et al. The non-canonical Wnt ligand, Wnt5a, is upregulated and associated with epithelial to mesenchymal transition in epithelial ovarian cancer. Gynecol Oncol. 2014. Epub 2014/06/14. doi: S0090-8258(14)01029-4 [pii] 10.1016/j.ygyno.2014.06.004 . 24924122

[20] Yoshioka S, King ML, Ran S, Okuda H, MacLean JA 2nd, McAsey ME, et al WNT7A regulates tumor growth and progression in ovarian cancer through the WNT/beta-catenin pathway. Mol Cancer Res. 2012;10(3):469–82. Epub 2012/01/11. 10.1158/1541-7786.MCR-11-0177 1541-7786.MCR-11-0177 [pii]. 22232518 PMC3307825

[21] Bast RC Jr, Feeney M, Lazarus H, Nadler LM, Colvin RB, Knapp RC. Reactivity of a monoclonal antibody with human ovarian carcinoma. J Clin Invest. 1981;68(5):1331–7. Epub 1981/11/01. 7028788 10.1172/JCI110380 PMC370929

[22] Yu D, Wolf JK, Scanlon M, Price JE, Hung MC. Enhanced c-erbB-2/neu expression in human ovarian cancer cells correlates with more severe malignancy that can be suppressed by E1A. Cancer Res. 1993;53(4):891–8. Epub 1993/02/15. . 8094034

[23] Joseph S, Deneke VE, Cowden Dahl KD. ARID3B induces tumor necrosis factor alpha mediated apoptosis while a novel ARID3B splice form does not induce cell death. PLoS One. 2012;7(7):e42159 Epub 2012/08/04. 10.1371/journal.pone.0042159 PONE-D-12-10269 [pii]. 22860069 PMC3409141

[24] Fogh J. Human Tumor Cells in vitro New York: Plenum Press; 1975.

[25] Hamilton TC, Young RC, McKoy WM, Grotzinger KR, Green JA, Chu EW, et al Characterization of a human ovarian carcinoma cell line (NIH:OVCAR-3) with androgen and estrogen receptors. Cancer Res. 1983;43(11):5379–89. Epub 1983/11/01. . 6604576

[26] Karlan BY, Jones J, Slamon DJ, Lagasse LD. Glucocorticoids stabilize HER-2/neu messenger RNA in human epithelial ovarian carcinoma cells. Gynecol Oncol. 1994;53(1):70–7. Epub 1994/04/01. doi: S0090825884710900 [pii]. . 7909787 10.1006/gyno.1994.1090

[27] Graham FL, Smiley J, Russell WC, Nairn R. Characteristics of a human cell line transformed by DNA from human adenovirus type 5. J Gen Virol. 1977;36(1):59–74. Epub 1977/07/01. . 886304 10.1099/0022-1317-36-1-59

[28] Choi JH, Choi KC, Auersperg N, Leung PC. Overexpression of follicle-stimulating hormone receptor activates oncogenic pathways in preneoplastic ovarian surface epithelial cells. J Clin Endocrinol Metab. 2004;89(11):5508–16. Epub 2004/11/09. doi: 89/11/5508 [pii] 10.1210/jc.2004-0044 . 15531506

[29] Wu Z, Irizarry RA. Stochastic models inspired by hybridization theory for short oligonucleotide arrays. J Comput Biol. 2005;12(6):882–93. Epub 2005/08/20. 10.1089/cmb.2005.12.882 . 16108723

[30] Tusher VG, Tibshirani R, Chu G. Significance analysis of microarrays applied to the ionizing radiation response. Proc Natl Acad Sci U S A. 2001;98(9):5116–21. Epub 2001/04/20. 10.1073/pnas.091062498 11309499 PMC33173

[31] Tamimi Y, Lines M, Coca-Prados M, Walter MA. Identification of target genes regulated by FOXC1 using nickel agarose-based chromatin enrichment. Invest Ophthalmol Vis Sci. 2004;45(11):3904–13. Epub 2004/10/27. doi: 45/11/3904 [pii] 10.1167/iovs.04-0628 . 15505035

[32] Bailey TL, Elkan C. Fitting a mixture model by expectation maximization to discover motifs in biopolymers. Proc Int Conf Intell Syst Mol Biol. 1994;2:28–36. Epub 1994/01/01. . 7584402

[33] Bailey TL, Boden M, Buske FA, Frith M, Grant CE, Clementi L, et al MEME SUITE: tools for motif discovery and searching. Nucleic Acids Res. 2009;37(Web Server issue):W202–8. Epub 2009/05/22. 10.1093/nar/gkp335 gkp335 [pii]. 19458158 PMC2703892

[34] Halperin Y, Linhart C, Ulitsky I, Shamir R. Allegro: analyzing expression and sequence in concert to discover regulatory programs. Nucleic Acids Res. 2009;37(5):1566–79. Epub 2009/01/20. 10.1093/nar/gkn1064 gkn1064 [pii]. 19151090 PMC2655690

[35] Linhart C, Halperin Y, Shamir R. Transcription factor and microRNA motif discovery: the Amadeus platform and a compendium of metazoan target sets. Genome Res. 2008;18(7):1180–9. Epub 2008/04/16. 10.1101/gr.076117.108 gr.076117.108 [pii]. 18411406 PMC2493407

[36] Carey MF, Peterson CL, Smale ST. Chromatin immunoprecipitation (ChIP). Cold Spring Harb Protoc. 2009;2009(9):pdb prot5279. Epub 2010/02/12. 10.1101/pdb.prot5279 2009/9/pdb.prot5279 [pii]. . 20147264

[37] Samyesudhas SJ, Roy L, Cowden Dahl KD. Differential expression of ARID3B in normal adult tissue and carcinomas. Gene. 2014;543(1):174–80. Epub 2014/04/08. 10.1016/j.gene.2014.04.007 S0378-1119(14)00407-7 [pii]. . 24704276

[38] Kim D, Probst L, Das C, Tucker PW. REKLES is an ARID3-restricted multifunctional domain. J Biol Chem. 2007;282(21):15768–77. Epub 2007/04/03. doi: M700397200 [pii] 10.1074/jbc.M700397200 . 17400556

[39] Tao J, Zhi X, Tian Y, Li Z, Zhu Y, Wang W, et al CEP55 contributes to human gastric carcinoma by regulating cell proliferation. Tumour Biol. 2014;35(5):4389–99. Epub 2014/01/07. 10.1007/s13277-013-1578-1 . 24390615

[40] Qiu JJ, Guo JJ, Lv TJ, Jin HY, Ding JX, Feng WW, et al Prognostic value of centromere protein-A expression in patients with epithelial ovarian cancer. Tumour Biol. 2013;34(5):2971–5. Epub 2013/05/29. 10.1007/s13277-013-0860-6 . 23712606

[41] Ricken A, Lochhead P, Kontogiannea M, Farookhi R. Wnt signaling in the ovary: identification and compartmentalized expression of wnt-2, wnt-2b, and frizzled-4 mRNAs. Endocrinology. 2002;143(7):2741–9. Epub 2002/06/20. 10.1210/endo.143.7.8908 . 12072409

[42] Barbolina MV, Burkhalter RJ, Stack MS. Diverse mechanisms for activation of Wnt signalling in the ovarian tumour microenvironment. Biochem J. 2011;437(1):1–12. Epub 2011/06/15. 10.1042/BJ20110112 BJ20110112 [pii]. 21668411 PMC3133882

[43] Gubbels JA, Belisle J, Onda M, Rancourt C, Migneault M, Ho M, et al Mesothelin-MUC16 binding is a high affinity, N-glycan dependent interaction that facilitates peritoneal metastasis of ovarian tumors. Mol Cancer. 2006;5(1):50. Epub 2006/10/28. doi: 1476-4598-5-50 [pii] 10.1186/1476-4598-5-50 17067392 PMC1635730

[44] Cannistra SA, Kansas GS, Niloff J, DeFranzo B, Kim Y, Ottensmeier C. Binding of ovarian cancer cells to peritoneal mesothelium in vitro is partly mediated by CD44H. Cancer Res. 1993;53(16):3830–8. Epub 1993/08/15. . 8339295

[45] Berns EM, Klijn JG, Henzen-Logmans SC, Rodenburg CJ, van der Burg ME, Foekens JA. Receptors for hormones and growth factors and (onco)-gene amplification in human ovarian cancer. Int J Cancer. 1992;52(2):218–24. Epub 1992/09/09. . 1325950 10.1002/ijc.2910520211

[46] Bartlett JM, Langdon SP, Simpson BJ, Stewart M, Katsaros D, Sismondi P, et al The prognostic value of epidermal growth factor receptor mRNA expression in primary ovarian cancer. Br J Cancer. 1996;73(3):301–6. Epub 1996/02/01. 8562334 10.1038/bjc.1996.53 PMC2074444

[47] Niikura H, Sasano H, Sato S, Yajima A. Expression of epidermal growth factor-related proteins and epidermal growth factor receptor in common epithelial ovarian tumors. Int J Gynecol Pathol. 1997;16(1):60–8. Epub 1997/01/01. . 8986534 10.1097/00004347-199701000-00010

[48] Scambia G, Benedetti Panici P, Battaglia F, Ferrandina G, Baiocchi G, Greggi S, et al Significance of epidermal growth factor receptor in advanced ovarian cancer. J Clin Oncol. 1992;10(4):529–35. Epub 1992/04/01. . 1548517 10.1200/JCO.1992.10.4.529

[49] Stewart CJ, Owens OJ, Richmond JA, McNicol AM. Expression of epidermal growth factor receptor in normal ovary and in ovarian tumors. Int J Gynecol Pathol. 1992;11(4):266–72. Epub 1992/10/01. . 1399232 10.1097/00004347-199210000-00004

[50] van der Burg ME, Henzen-Logmans SC, Foekens JA, Berns EM, Rodenburg CJ, van Putten WL, et al The prognostic value of epidermal growth factor receptors, determined by both immunohistochemistry and ligand binding assays, in primary epithelial ovarian cancer: a pilot study. Eur J Cancer. 1993;29A(14):1951–7. Epub 1993/01/01. . 8280488 10.1016/0959-8049(93)90451-k

