# Supplementary figures and images for: ARID3B Directly Regulates Ovarian Cancer Promoting Genes
Source: PLoS One. 2015 Jun 29;10(6):e0131961. doi: 10.1371/journal.pone.0131961 (PMC4486168; doi:10.1371/journal.pone.0131961)

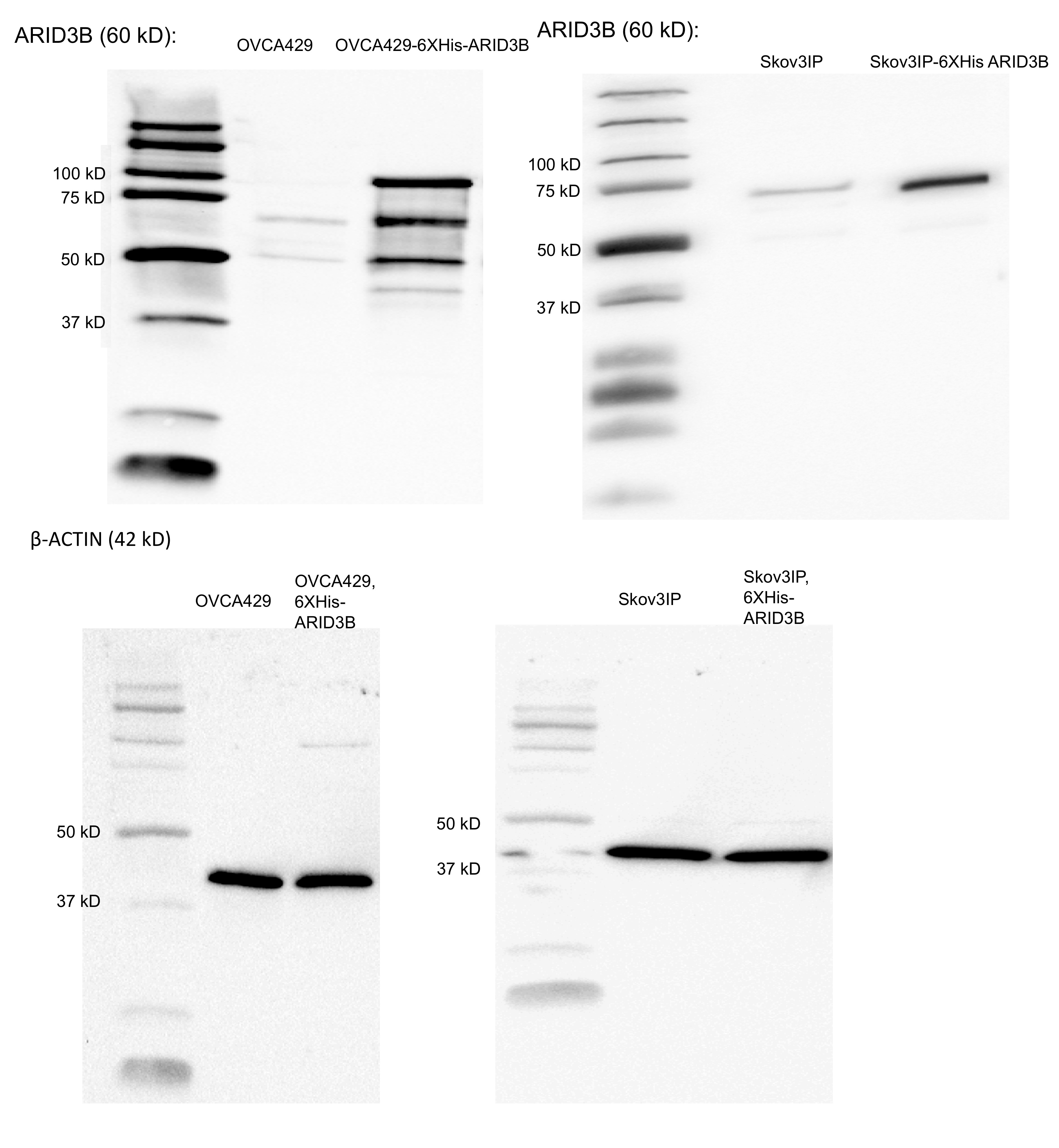

Supplement: S1 Fig — Uncropped western blot showing detection of ARID3B in parental OVCA429 and Skov3IP cell lines compared to the cell lines transduced with 6XHis-ARID3B, as shown in Fig 1. Uncropped blot for β-Actin loading controls is also included. (TIF) [file pone.0131961.s001.tif]

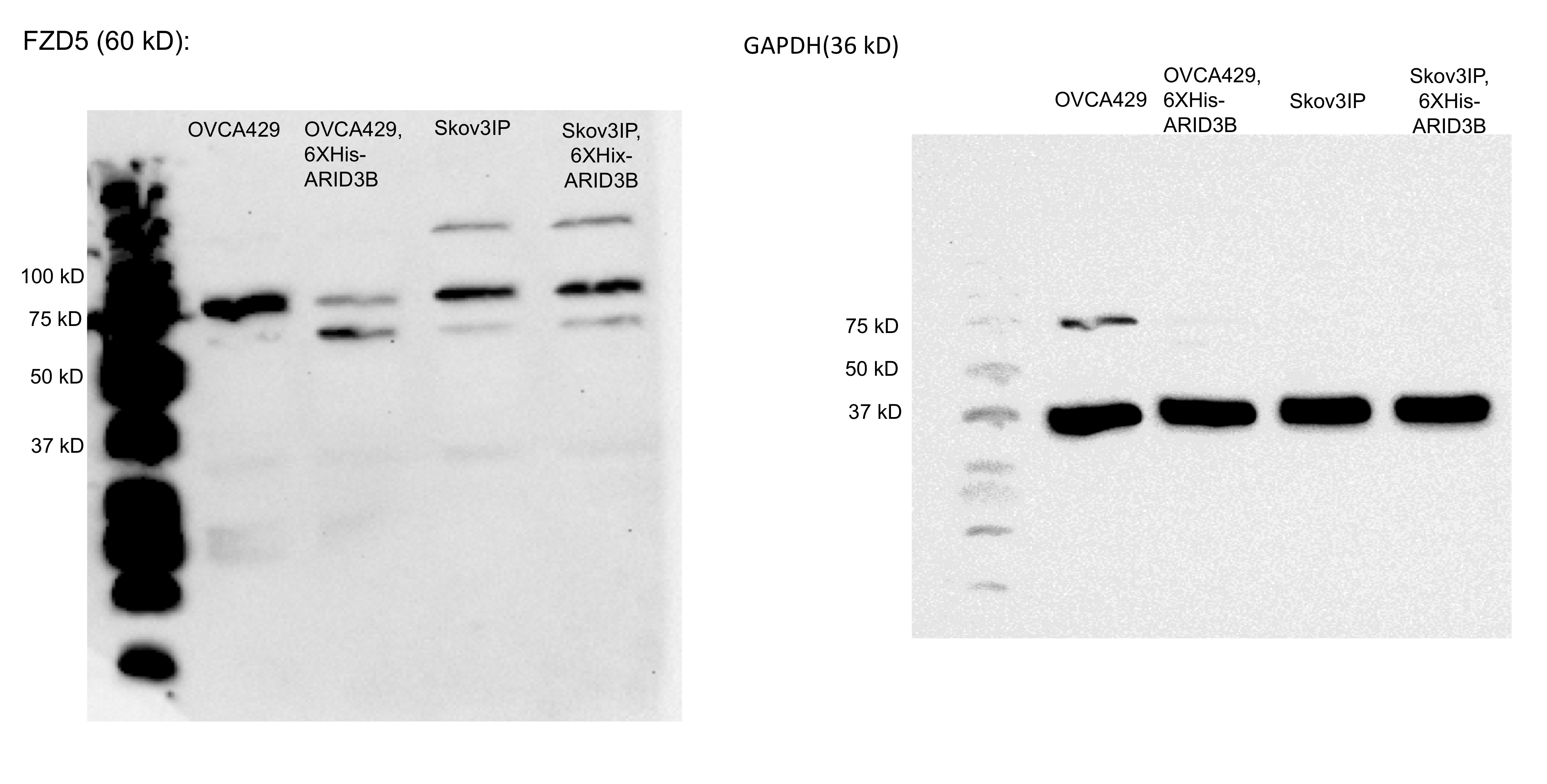

Supplement: S2 Fig — Uncropped western blot showing detection of FZD5 in parental OVCA429 and Skov3IP cell lines compared to cell lines transduced with pGMP-FZD5, as shown in Fig 4. Uncropped blot for GAPDH loading controls. (TIF) [file pone.0131961.s002.tif]

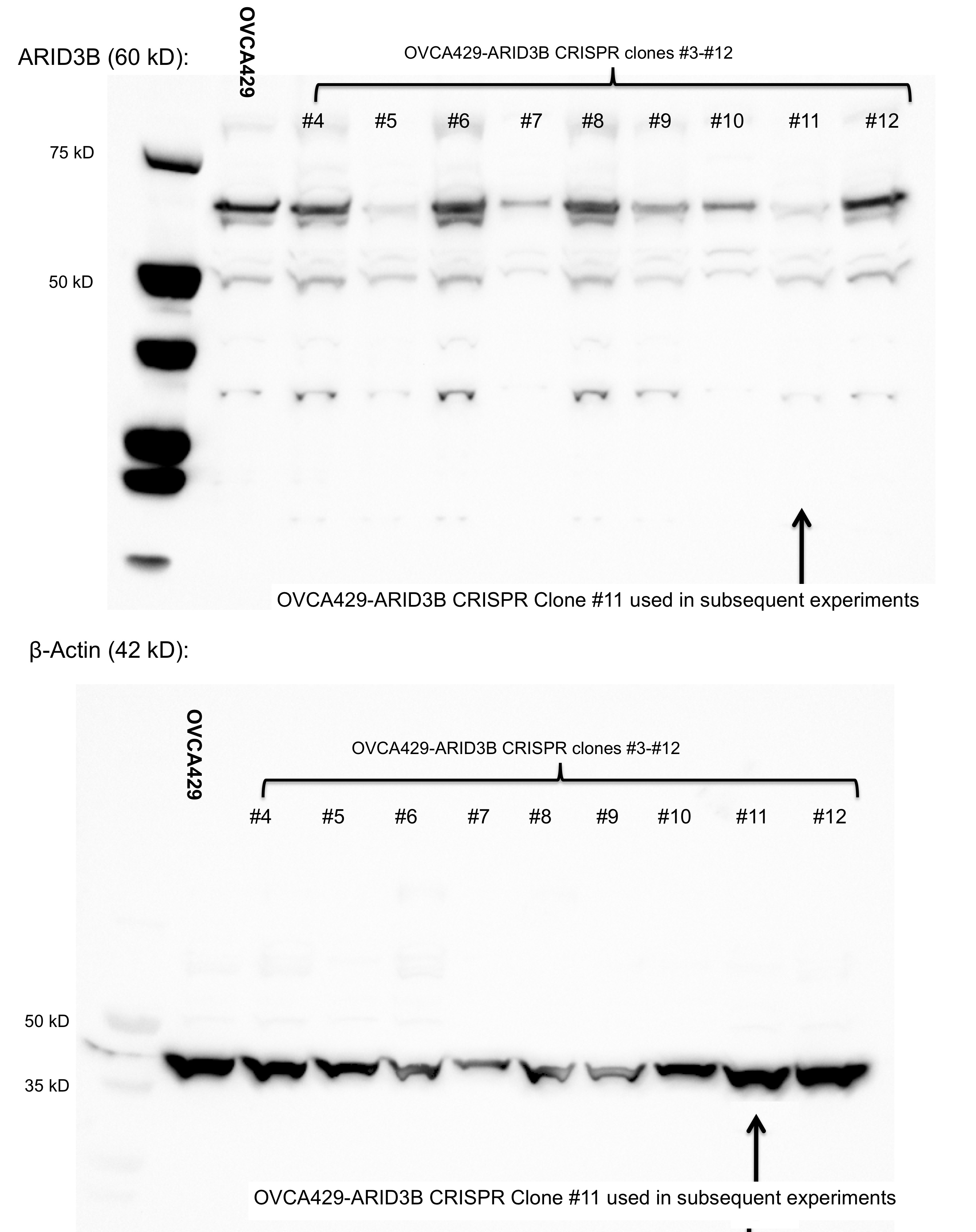

Supplement: S3 Fig — Uncropped western blot for ARID3B in OVCA429 parental cells, versus OVCA429 cells edited with a CRISPR sgRNA targeting ARID3B, as shown in Fig 5. Samples loaded in other lanes were not used in subsequent experiments. Uncropped blot for β-Actin loading controls. (TIF) [file pone.0131961.s003.tif]

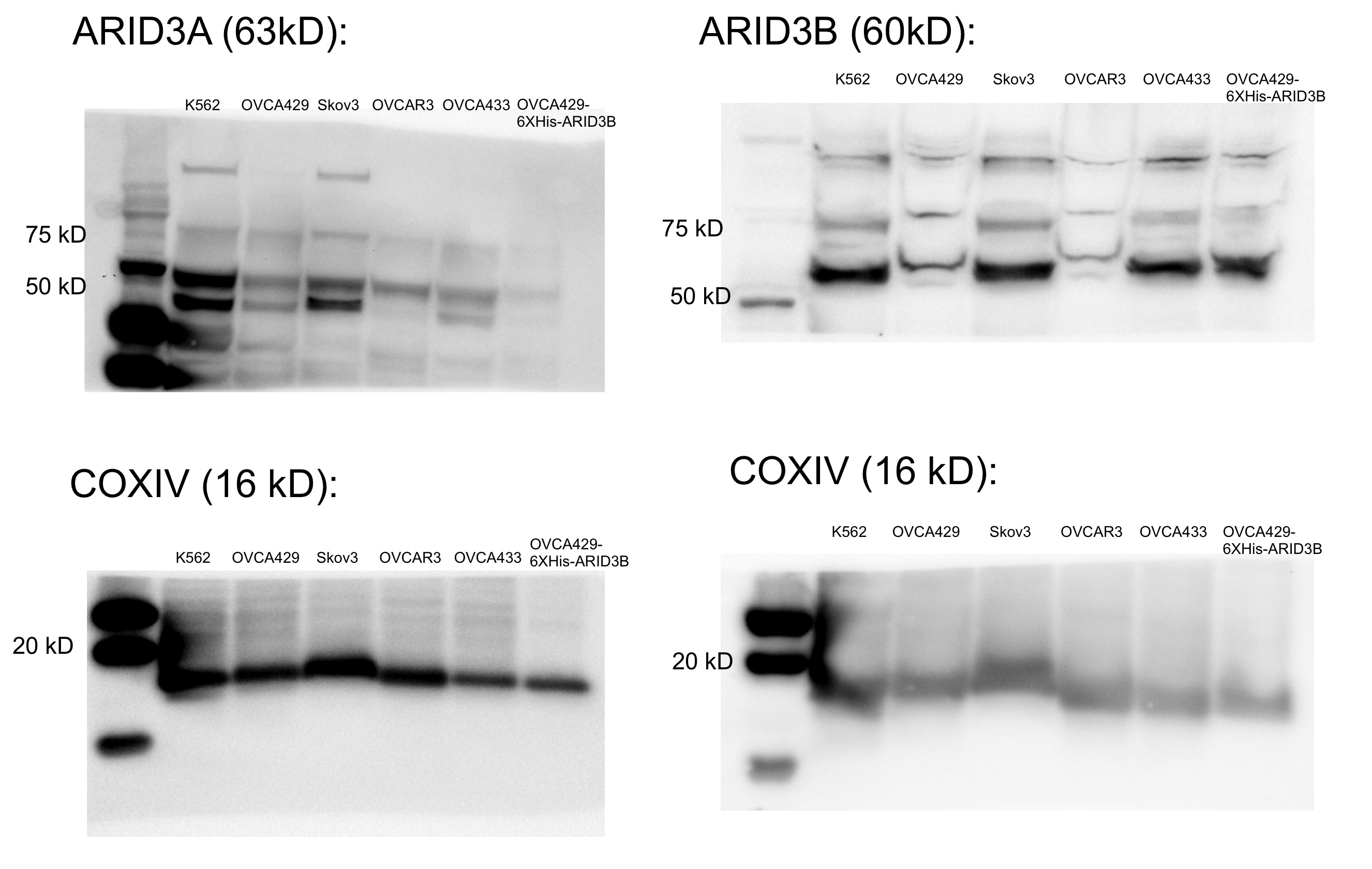

Supplement: S4 Fig — Uncropped western blot for ARID3B and ARID3A in various ovarian cancer cell lines, as shown in Fig 7. Uncropped blot for COXIV loading control. (TIF) [file pone.0131961.s004.tif]
